# Fertility desires of people living with HIV: does the implementation of a sexual and reproductive health and HIV integration model change healthcare providers’ attitudes and clients’ desires?

**DOI:** 10.1186/s12913-021-06487-0

**Published:** 2021-05-26

**Authors:** Cecilia Milford, Mags Beksinska, Ross Greener, Jacqueline Pienaar, Letitia Rambally Greener, Zonke Mabude, Jennifer Smit

**Affiliations:** 1 MRU (MatCH Research Unit), Department of Obstetrics and Gynaecology, Faculty of Health Sciences, University of the Witwatersrand, Commercial City Building, 40 Dr AB Xuma Street, Durban, 4001 South Africa; 2 The Aurum Institute, Johannesburg, South Africa; 3 The Centre for HIV-AIDS Prevention Studies, Johannesburg, South Africa

**Keywords:** HIV positive, PLHIV, Fertility desires, Provider, Client, Integration, South Africa

## Abstract

**Background:**

There is a need for information and healthcare support for the fertility desires and contraceptive needs of people living with HIV (PLHIV) in order to provide safer conception support for sero-discordant couples wanting to safely conceive. A model to integrate sexual and reproductive health and HIV services was developed and implemented in a district hospital and six clinics in the eThekwini District, South Africa.

**Methods:**

To evaluate the model’s success, a cross-sectional survey was conducted before and after implementation of the model. As part of this evaluation, fertility desires of PLHIV (both male and female), and providers’ perspectives thereof were explored. Changes in desires and attitudes after integration of services were investigated.

**Results:**

Forty-six healthcare providers and 269 clients (48 male, 221 female) were surveyed at baseline, and 44 providers and 300 clients (70 male, 230 female) at endline. Various factors including relationship status, parity and antiretroviral treatment (ART) access influenced PLHIVs’ desires for children. Concerns for their own and their child’s health negatively impacted on PLHIV’s fertility desires. These concerns declined after integration of services. Similarly, providers’ concerns about PLHIV having children decreased after the implementation of the model.

**Conclusions:**

Integrated services are important to facilitate provision of information on contraceptive options as well as safer conception information for PLHIV who want to have children.

**Supplementary Information:**

The online version contains supplementary material available at 10.1186/s12913-021-06487-0.

## Background

South Africa has the world’s largest population of people living with HIV (7.1 million in 2016; 19% of the global number of people living with HIV) [1], with the burden of HIV disproportionately affecting young women [2]. According to the South African Demographic and Health Survey (2016), there was a total fertility rate of 2.6 children per woman aged 15–49 years in the country in 2016 [2]. The fertility rate in South Africa is lower than in other African countries and has decreased over time due to factors including: rapid social and economic development, and access to and acceptability of contraception [3]. Despite this, there are still high rates of unplanned pregnancies [4], with 18% of women having an unmet family planning need [2].

People living with HIV (PLHIV) also have reproductive needs and require access to information and quality family planning services to prevent unintended pregnancies and to facilitate safe and healthy pregnancies [5]. They require access to reliable contraception so that they can have only the number of children that they desire (reproductive choice), and need support and access to perinatal mother-to-child transmission (PMTCT) services to minimize the chances of mother-to-child transmission [6]. PLHIV, especially adolescents in developing countries, often do not have sufficient information about contraception and parenting options [7]. In particular, there is a need for attention to be paid to current fertility desires and contraceptive needs of couples affected by HIV and to provide safer conception support for sero-discordant couples wanting to safely conceive.

Lack of service integration has been identified as one of the main reasons that the sexual and reproductive health (SRH) needs of PLHIV remain unmet [8]. There are numerous challenges to integrating services in a developing world context, like Southern Africa. Many of these are associated with understaffed healthcare facilities, lack of physical infrastructure/space, commodity shortages and a need for additional training of healthcare providers [9, 10]. The lack of resources (infrastructural and human), and difficult working conditions, can lead to poor motivation of healthcare providers, which in turn can hamper service integration [11]. However, implementing integrated SRH programs can address peri-conception risk [12], and can assist with appropriate pregnancy planning among HIV affected couples. Integration of services is important to facilitate information provision so that PLHIV can make informed SRH decisions [13, 14]. PLHIV need to be provided with information on potential drug interactions, for example the possible decreased efficacy of hormonal implant when used together with efavirenz (ART) [15], as well as be informed about facts, such as the teratogenic effect of Dolutegravir (first line antiretroviral regimen) on babies [16]. Furthermore, the integration of SRH and family planning into HIV services creates a less stigmatizing environment for PLHIV to discuss fertility desires, contraception and sexuality with healthcare providers [17].

Research has demonstrated that both men and women living with HIV desire to have children [18–20]. Fertility desires in couples both affected and unaffected by HIV are influenced by various factors, including education and economic opportunities, decision making relations with partners, family and community pressure, and parity [3, 20–23]. In one study, people in more stable relationships were found to be likely to want more children [20], and another described a cultural expectation for proving fertility in long term relationships [24]. It has also been found that women with fewer children have a greater desire for children in future [3, 20, 21].

Knowledge of HIV status has also been found to influence fertility desire, and a study in Malawi demonstrated that the number of women wanting children declined after testing positive for HIV [21]. This could be related to concerns of poor health for self and child [20, 22, 24], leaving a child orphaned, and financial concerns [22]. Partner HIV status may also impact on fertility desires – females with HIV positive partners may be less likely to desire pregnancy or want more children [3]. However, availability of treatment (highly active antiretroviral therapy (HAART) and prevention of mother to child transmission (PMTCT)) has also impacted on fertility desires [3, 20, 25, 26], and research has shown that women with access to antiretroviral treatment (ART) may have greater desires for children [26]. There are also concerns about interaction of HIV treatment and contraceptive methods [27], resulting in switching or stopping method use. There is limited data on fertility desires in HIV positive males, and on factors influencing their desires for children.

There are supportive guidelines in South Africa recommending that safer conception counselling be part of routine HIV care [28, 29], which intend to provide PLHIV with options for achieving their fertility desires. South African policies promote the SRH rights of PLHIV and promote provider-initiated discussions on reproductive goals for PLHIV [30, 31]. Despite these policies and guidelines, there are implementation challenges related to providers having limited information about safer conception options as well as concerns about discussing safer conception with HIV affected couples [30–33]. Some providers have described ethical conflict in providing safer conception counselling, especially where poverty and intimate partner violence are concerns [12]. In addition to providing knowledge and support, it is critical that there is political will to promote strategies and interventions [13], especially to facilitate implementation of the supportive guidelines.

The perceived attitudes and views of healthcare providers towards reproductive choice in HIV infected women and men are important, as their views can influence clients’ reproductive decision-making [20, 34]. A study in India, Cambodia, Indonesia, Nepal, Bangladesh and Vietnam found that despite the availability of integrated services, many doctors were unsupportive of positive women’s desires to have children, making it difficult for them to get advice on this [35].

Integration of SRH and HIV services is a complex but essential step forward to enable improvement of health outcomes. Although some PLHIV do not wish to have children, some are having/have had children, and some women and their male partners want to have children in the future. These women, men and couples need information on how to prevent pregnancies and how to have children safely, from healthcare providers who will not judge them.

The purpose of this manuscript is to present findings on the fertility desires of HIV positive women and men attending seven public health sector facilities in the eThekwini District of KwaZulu-Natal, South Africa. We also explore perspectives of healthcare providers, working in the same facilities, about the reproductive desires of PLHIV. Client fertility desires and provider perspectives are described before and after the implementation of a model for integrating SRH and HIV services, and changes in these are also described.

## Methods

This study was conducted in a district hospital and six of its feeder clinics in the eThekwini District, KwaZulu-Natal, South Africa between 2009 and 2011. The study site was chosen based on discussions with the Department of Health (DoH) who recommended that the sub-district was in need of systemic and structural changes and that it would benefit from an integration study at the time of the project. The sites selected reflect the way in which service delivery is provided in the District.

**Fig. 1:**
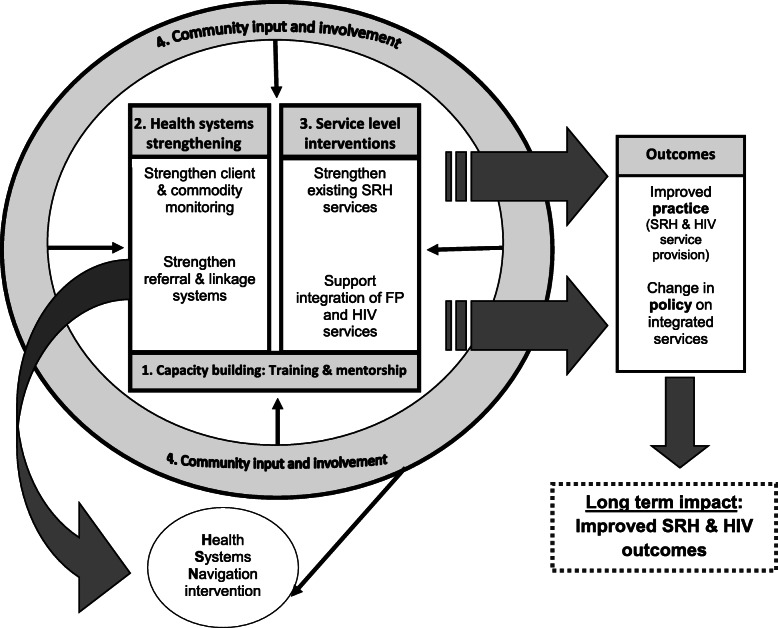
Conceptual representation of SRH services integration model [37]

In 2009, baseline data were collected using a facility audit and cross-sectional survey. This was done in order to better understand the SRH, family planning and HIV services offered at these facilities, as well as the status of integration of these services in and between these facilities. The baseline data were used to inform the development of a district-based model for integrating SRH and HIV services, which was implemented in 2010 at the hospital and feeder clinics [36–38] (Fig. 1).

The integration model [37] focused on health systems strengthening and strong community input and involvement. The principle of health systems strengthening included measures to improve information and supply chains through improved patient and commodity monitoring, and through innovative methods to improve referral and linkage systems. This was done by strengthening referrals both within and between healthcare facilities and by making use of a novel “health navigation” strategy [39]. Existing family planning (FP) and SRH services were targeted for integration, and rather than creating a generic package, the model responded to particular needs of individual facilities. FP services were introduced into ART clinics, HIV wellness centres, well baby (postnatal clinics) and PMTCT clinics, and HIV testing and counselling was conducted by FP service providers. Primary healthcare (PHC), antenatal care (ANC), sexually transmitted infection (STI) and termination of pregnancy (TOP) services were also included. In addition, capacity building for healthcare providers was done via focused training and mentorship programs. Healthcare provider training was based on needs identified at the facilities in this project, and focused on various modules including referral systems, HIV counselling and testing (HCT) and FP integration, ART and FP integration, antiretroviral (ARV) and FP method interactions, dual protection against HIV and unplanned pregnancy, and comprehensive care and management of HIV clients. Health systems navigators, as peer supporters [40], were also trained to provide education and support on these topics at community and health facility level. The development and implementation of the model, its successes and challenges, is described in detail elsewhere [37]. This was a pilot study to inform the development of an integration model which could be rolled out and evaluated by local and national health departments.

There was strong stakeholder and community involvement [37]. A Community Advisory Board was established, and key stakeholders from health facilities and the DoH (Provincial and District level) were consulted at regular time points, prior to, during and after, the model implementation process. These community and stakeholder interactions provided a platform for them to provide input on the model design and implementation. They were also given opportunities to participate in training and health-promotion activities.

The model was implemented in a phased fashion (executed in a staggered/step-wise manner), with activities occurring until the end of 2011. For example, healthcare provider training was conducted in modules, and the same modules were conducted in different facilities at similar times. As a result, exposure did not differ greatly between facilities. After the implementation of the model, endline cross-sectional data were collected and a facility audit was conducted at the end of 2011, on the same key variables of interest as the baseline studies, to be able to compare findings. This comparison was important to be able to determine the usefulness and successes of the model. One of the clinics dropped out of the intervention, due to perceived lack of time to participate in intervention activities, and therefore only the district hospital and five feeder clinics participated in the endline survey. This manuscript focuses on relevant data collected as part of the cross-sectional surveys. Data from other aspects of the study are reported elsewhere [36–38].

### Study population

For the baseline cross-sectional survey, the study population comprised 46 healthcare providers (41 female, 5 male) working at, and 269 clients (221 female, 48 male), attending the seven healthcare facilities. For the endline cross-sectional survey, there were 44 providers (41 female, 3 male) working at, and 300 clients (230 female, 70 male) attending the six healthcare facilities.

Potential healthcare provider participants were identified by purposively selecting providers representing different categories of healthcare provision (including HCT counselors, enrolled nurses, registered nurses and doctors). They were identified from registers of providers working in the participating healthcare facilities, who were available on the day of the interview. They were purposively sampled from different service points in these facilities, including ANC, FP, PHC, HCT, TOP and STI service points, if available, and were invited to participate. If they were not willing or unable to participate due to high workloads, alternative staff of that cadre were invited to participate. Purposive selection was done to make certain that there would be a range of provider types interviewed across a range of services, and this was done to ensure that study results would not be biased according to service or type of provider. Sample size was limited by the number of providers working in the relevant service areas in the participating facilities.

The client sample was selected by ensuring representation of clients attending the different SRH services in the designated facilities. Where low numbers of clients were attending a particular service, additional clients were purposively sampled, in order to interview at least one client per service. This purposive selection included targeting clients attending HIV treatment and care services, therefore the proportion of HIV infected participants in the total client sample may not be representative of HIV statistics in the region. Higher numbers of clients were recruited from HIV/ARV services at endline than at baseline, possibly due to more clients attending these services at endline. Clients were approached when they were leaving the facility to participate in exit interviews. We aimed to interview approximately 30–40 clients per clinic, and approximately 50 clients at the hospital, to ensure a total sample size of between 250 and 300 clients.

The sample size was determined based on budget, time constraints and staffing available across the sites, using both purposive and convenience sampling methods in an attempt to obtain representation of providers and clients accessing services at the designated facilities. This sampling strategy was appropriate for the exploratory nature of the study.

### Data collection and analysis

Participants answered structured survey questionnaires which were developed specifically for this study (see attached supplementary documents – provider baseline and endline questionnaires, and client baseline and endline questionnaires). The provider interview schedule focused on training, resources, delivery of services, and values, and providers were asked about their perceptions of pregnancy in HIV positive women. The client interview schedule asked details about the type and quality of service they received, their HIV status and FP needs. All clients were asked: “Do you want to have more children?”. Due to the fact that some female clients were attending ANC services, we cannot be sure if their responses about desire for more children referred to future pregnancies or included their current pregnancy, although where possible interviewers asked them to consider future pregnancies. Male and female clients who disclosed that they were HIV positive were further asked about their future fertility desires and how their HIV status had affected their decisions about future pregnancies.

This manuscript explores one component of this cross-sectional survey; HIV infected clients’ desires for more/no more children, and compares these desires with select demographic details. All participants over 45 years of age are excluded in the analysis, as fertility rates after age 45 tend to be low [2] for various reasons, including biological ability to have children. Healthcare providers’ perspectives of PLHIV having children are also explored in this manuscript.

Data were captured into and descriptively analysed using SPSS v25. Given the limitations to the sample selection and the cross-sectional nature of the survey Pearsons’chi-square square or Fisher’s exact test of association (where sample size was small) were calculated for some categorical variables to tentatively explore statistical differences between baseline and endline data (*p* < 0.05).

### Ethical considerations

The study was reviewed and approved by the University of Witwatersrand’s Human Research Ethics Committee (HREC) (reference number M080624), and reciprocity approval was granted by the University of KwaZulu-Natal’s Biomedical Research Ethics Committee (BREC). Each participant completed individual informed consent to enable the research team to collect questionnaire data. No medical records or any other records at Department of Health facilities were accessed. Permission to conduct the research in the facilities was granted by each participating healthcare facility and additionally from the KwaZulu-Natal Provincial, eThekwini District and Municipal DoH. The University of the Witwatersrand HREC approved all the consent documents as did the DoH.

## Results

### Client sample

Male and female clients were sampled from various health service points, and may have been accessing more than one service on the day of the survey (Table 1). At baseline, the highest proportion of clients sampled were accessing PHC, immunization, and ANC follow-up care, compared with HIV/ART, PHC and ANC follow-up care at endline.

**Table 1:** Client profile: Proportion of clients accessing different services at baseline and endline

Services attended^a^	Baseline ***n*** = 269 (%)	Endline ***n*** = 300 (%)
HIV/ART clinic	39 (14.5)	101 (33.7)
PHC	67 (24.9)	97 (32.3)
HCT	32 (11.9)	42 (14.0)
Immunization/ Postnatal care (PNC)	45 (16.7)	27 (9.0)
ANC follow up	43 (15.9)	50 (16.7)
ANC 1st visit	13 (4.8)	7 (2.3)
Family planning	35 (13.0)	27 (9.0)
STI	2 (0.7)	10 (3.3)
TOP	7 (2.6)	0

Abbreviations: *HIV/ART* Human Immunodeficiency Virus/antiretroviral treatment, *PHC* primary healthcare, *HCT* HIV counselling and testing, *ANC* antenatal care, *STI* sexually transmitted infection, *TOP* termination of pregnancy

^a^Some participants presented for multiple services

### HIV infected clients’ desires for having children in future

At baseline, a third (*n* = 86, 31.9%) of clients self-reported that they were HIV positive, of which 14 (16%) were male and 72 (84%) were female. At endline, just under half of the clients (*n* = 144, 48%) self-reported that they were HIV positive, and of these 43 (30%) were male, and 101 (70%) female. At baseline, 24 (8.9%) participants, and at endline, 30 (10%) participants, did not disclose their HIV results. Fertility desires are described only for those HIV positive clients (men and women) who were aged 45 years or less. Table 2 highlights the proportions of HIV positive males and female clients overall, and then more specifically, those who were aged 45 years or less, and their desires for children.

**Table 2:** HIV infected male and female’s desires for children

	Sex	All HIV positive (n)	45 years or younger and HIV positive (n)	Want to have more children		Do not want /unsure if want to have more children	
				All HIV positive n (%)	45 years or younger n (%)	All HIV positive n (%)	45 years or younger n (%)
**Baseline**	**Female**	72	65	10 (13.9)	10 (15.4)	62 (86.1)	55 (84.6)
	**Male**	14	11	6 (42.9)	6 (54.5)	8 (57.1)	5 (45.5)
**Endline**	**Female**	101	95	37 (36.7)	37 (38.9)	64 (63.3)	58 (61.1)
	**Male**	43	38	18 (41.9)	17 (44.7)	25 (58.1)	21 (55.3)

Looking specifically at HIV positive participants 45 years or younger, at both baseline and endline, a higher proportion of HIV positive males than females wanted more children, and although the proportion of females wanting more children increased between baseline and endline, the proportion of males decreased. Pearson’s chi-square test demonstrated that the difference between baseline and endline desires for more children (in the group of 45 years and less) was statistically significant (*p* = 0.001).

Current pregnancy status may have impacted expressed desires for children (as noted in the section on data collection and analysis). Eleven of the 65 HIV positive women (45 years or younger) who didn’t want more children at baseline and nine out of 95 (HIV positive, 45 years or younger) at endline were attending ANC services (therefore currently pregnant).

### Characteristics of HIV positive clients and desires for children

Table 3 demonstrates characteristics of HIV infected clients and their desire for children.

**Table 3:** Characteristics of HIV positive clients and their desires for children

	Baseline				Endline				Significance of change over time	
	Positive Male ***n*** = 11		Positive Female ***n*** = 65		Positive Male ***n*** = 38		Positive Female ***n*** = 95			
	Want more children, ***n*** = 6 (54.5%)	Do not want more children/ not sure, ***n*** = 5 (45.5%)	Want more children, ***n*** = 10 (15.4%)	Do not want more children/ not sure, ***n*** = 55 (84.6%)	Want more children, ***n*** = 17 (44.7%)	Do not want more children/not sure, ***n*** = 21 (55.3%)	Want more children, ***n*** = 37 (38.9%)	Do not want more children/not sure, ***n*** = 58 (61.1%)	Chi-square ***P*** value Male	Chi-square ***P*** value Female
**Relationship status**									.428	.168
Married	0	1 (20.0)	2 (20.0)	8 (14.5)	1 (5.9)	1 (4.8)	6 (16.2)	3 (5.1)		
Regular partner	5 (83.3)	4 (80.0)	8 (80.0)	37 (67.3)	13 (76.5)	19 (90.5)	25 (67.6)	43 (74.1)		
Single	0	0	0	10 (18.2)	3 (17.6)	1 (4.8)	4 (10.8)	11 (19.0)		
Multiple partners/other	1 (16.7)	0	0	0	0	0	2 (5.4)	1 (1.7)		
**Partner’s HIV status**									.741	.870
Positive	4 (66.7)	2 (40.0)	4 (40.0)	18 (32.7)	5 (29.4)	11 (52.4)	9 (24.3)	18 (31.0)		
Negative	1 (16.7)	1 (20.0)	2 (20.0)	10 (18.2)	3 (17.6)	3 (14.3)	7 (18.9)	10 (17.2)		
Did not disclose	1 (16.7)	2 (40.0)	4 (40.0)	27 (49.1)	9 (52.9)	7 (33.3)	21 (56.8)	30 (51.7)		
**Number of children**									.003*	.171
None	1 (16.7)	1 (20.0)	2 (20.0)	6 (10.9)	0	0	0	0		
One	2 (33.3)	1 (20.0)	5 (50.0)	15 (27.3)	8 (47.1)	2 (9.5)	8 (21.6)	4 (6.9)		
Two	3 (50.0)	1 (20.0)	3 (30.0)	20 (36.4)	5 (29.4)	2 (9.5)	18 (48.6)	21 (36.2)		
Three	0	2 (40.0)	0	9 (16.4)	2 (11.8)	6 (28.6)	7 (18.9)	13 (22.4)		
Four or more	0	0	0	5 (9.1)	2 (11.8)	11 (52.4)	4 (10.8)	20 (34.5)		
**Age (years)**									.236	.001*
18–20	0	0	1 (10.0)	0	1 (5.9)	0	1 (2.9)	1 (1.7)		
21–25	1 (16.7)	1 (20.0)	4 (40.0)	10 (18.2)	5 (29.4)	0	15 (40.5)	8 (13.8)		
26–30	2 (33.3)	1 (20.0)	3 (30.0)	14 (25.5)	2 (11.8)	6 (28.6)	10 (27.0)	13 (22.4)		
31–35	2 (33.3)	1 (20.0)	1 (10.0)	19 (34.5)	4 (23.5)	6 (28.6)	8 (21.6)	13 (22.4)		
36–40	1 (16.7)	1 (20.0)	1 (10.0)	8 (14.5)	3 (17.6)	6 (28.6)	3 (8.1)	15 (25.9)		
41–45	0	1 (20.0)	0	4 (7.3)	2 (11.8)	3 (14.3)	0	8 (13.8)		
**Using method to prevent pregnancy**									.161	.000*
Yes	6 (100)	5 (100)	5 (50.0)	29 (52.7)	13 (76.5)	20 (95.2)	17 (45.9)	37 (63.8)		
No	0	0	3 (30.0)	20 (36.4)	1 (5.9)	1 (4.8)	3 (8.1)	4 (6.9)		
Not sexually active	0	0	0	6 (10.9)	3 (17.6)	0	0	8 (13.8)		
Currently pregnant	N/A	N/A	2 (20.0)	0	N/A	N/A	17 (45.9)	9 (15.5)		
**On antiretroviral treatment (ART)**^**a**^									.437**	1.000**
Yes	4 (80.0)	4 (80.0)	0	15 (50.0)	13 (92.8)	15 (78.9)	14 (73.6)	35 (85.4)		
No	1 (20.0)	1 (20.0)	1 (100)	15 (50.0)	1 (7.2)	4 (21.1)	5 (26.3)	6 (14.6)		

^a^Although there was a question asking whether clients were taking antiretrovirals (ARVs), not all responded to this question, therefore n is not the same as the other categories

*where *p* < 0.05 is significant

**Fisher’s exact test of association used, results not significant

#### Relationship status

Most HIV positive women and men reported having a regular partner. At both baseline and endline, the majority who wanted more children had a regular partner. Relationship status had no statistical significance.

#### Partner’s HIV status

The proportion of positive men and women who had HIV infected partners and wanted more children decreased from baseline to endline. Partner’s HIV status had no statistical significance.

#### Number of current children

At baseline, only HIV positive men and women who had less than three children wanted more children. At endline, some HIV positive men and women who had three or more children wanted more children, although this was outweighed by those with three or more children who didn’t want more children. There was a significant difference between baseline and endline with HIV positive men’s current number of children and desires for more children (*p* = 0.003).

#### Age of respondent

Female respondents ranged in age from 19 to 59 years, and males from 20 to 58 years. However, these data look only at men and women 45 years or younger. Higher proportions of younger (than older) HIV positive women and men, at both baseline and endline wanted more children. There was a significant difference between age and desires for more children of women between baseline and endline (*p* = 0.001).

#### Current contraceptive method use

Contraceptives were used both by those who wanted more children and those who did not want children in the future. The proportion of women who did not want children in future, using contraception, increased at endline, and this change was significant (*p* < .001).

#### Fertility desire and current use of ART

The desire to have more children (or not) was compared with whether clients were currently taking ARVs. At baseline, only one HIV positive woman who wanted more children responded to the question whether s/he was on ART, and she was not taking ARVs, therefore the change between baseline and endline for women is not easily comparable. However, there was an increase in the proportion of men on ART who wanted more children between baseline and endline, although this change was not significant.

### Factors affecting future fertility desires

HIV infected clients were asked about various issues that may have affected their future fertility desires (Table 4). They were given a list of issues which may have affected their fertility desires and asked which applied to them. The most common issues affecting fertility desires among clients *who wanted more children* at baseline were that they were still waiting for advice on the best time to fall pregnant (*n* = 5), and that they were worried about their own health (*n* = 4) more than that of their child’s health (*n* = 2). In contrast, at endline, more clients who wanted children were worried about the health of their child (*n* = 14) versus their own health (*n* = 9).

**Table 4:** Factors affecting fertility desires of male and female clients. (where applicable, the number of participants who were attending antenatal clinic (ANC) services and therefore pregnant, but whose responses are included in the data, is noted)

Factors affecting fertility desires	Baseline		Endline		Change over time	
	Want more children, ***n*** = 16 (%)	Do not want more children, ***n*** = 60 (%)	Want more children, ***n*** = 54 (%)	Do not want more children, ***n*** = 79 (%)	Want more children, ***p*** value**	Do not want more children, ***p*** value**
Been told not to have them	1 (6.3)	4 (6.7)	1 (1.9)	0	.407	.032*
Worried that child may get sick/die	2 (12.5)	25 (41.7) (5 attending ANC)	14 (25.9) (6 attending ANC)	24 (30.4) (3 attending ANC)	.329	.158
Worried about own health	4 (25.0) (2 attending ANC)	19 (31.7) (5 attending ANC)	9 (16.7) (4 attending ANC)	9 (11.4) (1 attending ANC)	.476	.005*
Waiting for viral load to decrease	1 (6.3)	N/A^a^	8 (14.8) (4 attending ANC)	N/A^a^	.673	N/A
Knowledge of status hasn’t changed anything	2 (12.5) (1 attending ANC)	0	14 (25.9) (4 attending ANC)	28 (35.4) (2 attending ANC)	.329	.000*
Still getting advice on best time to fall pregnant	5 (31.3) (2 attending ANC)	N/A^b^	11 (20.4) (6 attending ANC)	2 (2.5)^b^	.498	.507
Have child/ren already	N/A^c^	6 (10.0) (1 attending ANC)	1 (1.9) (this 1 also attending ANC)	11 (13.9)	1.00	.606

^a^Question was not asked of HIV positive women who did not want more children at baseline or endline

^b^Question was not asked of HIV positive women who did not want more children at baseline, but was asked at endline

^c^Question was not asked of HIV positive women who wanted more children at baseline, but was asked at endline

*where *p* < 0.05 is significant

**Fisher’s Exact test of association

Factors affecting fertility desires in clients *who did not want more children* at baseline were (1) that their child may get sick/die (*n* = 25); (2) concerns about their own health (*n* = 19), and (3) some were influenced by the fact that they had children already (*n* = 6). At endline, clients who did not want any more children were more concerned that their children may get sick/die (*n* = 24) than their own health (*n* = 9), and a few had children already (*n* = 14). At endline there were a large portion of clients who felt that the knowledge of their status did not affect their desire for having more children (*n* = 42).

There was a significant difference between baseline and endline for those clients who did not want more children, and who had been told not to have more children (*p* = 0.032), at endline no participants who had been told not to have more children, did not want any more children (Table 4). Concerns about own health were also significantly different between baseline and endline for those clients who did not want more children (and decreased over time) (*p* = 0.005). In addition, there was a significant difference between baseline and endline of those clients who did not want more children, who felt that the knowledge of their status had not changed their desire for children (*p* < .001).

### Provider sample

Healthcare providers were sampled from a variety of service delivery points and may have been working across multiple departments, providing multiple services. At baseline, the highest proportion of providers sampled were working in ANC (*n* = 20, 43.5%), PHC (*n* = 19, 41.3%) and PMTCT (*n = *19, 41.3%) services, compared with PMTCT (*n* = 17, 38.6%), HCT (*n* = 16, 36.4%) and ANC (*n* = 14, 31.8%) services at endline. Others worked in postnatal, FP, STI, TOP and ART services.

### Providers’ perceptions of HIV positive women and pregnancy

Providers were asked for their views on whether/when HIV positive women should consider having children. At baseline, 25 (54.3%) providers thought that healthy HIV positive women should have children if they so desired, compared with 28 (63.6%) at endline. One other provider (2.2%) at baseline felt it would be appropriate for HIV positive women to have children if they had no children already. At endline, a further two (4.5%) providers felt that these women should have children if they are healthy/have a high CD4. At baseline, 11 (23.9%) providers did not think that HIV positive women should have children, in contrast to only three (6.8%) at endline.

When exploring attitudes around PLHIV having children, at baseline most providers (*n* = 35, 87%) did not agree that pregnant HIV positive women should have an abortion, which increased at endline to 93.2% (*n* = 41). Furthermore, at baseline half of the providers (*n* = 23) felt that women on ART should not fall pregnant, which decreased at endline to only 18.2% (*n* = 8). Finally, many providers at baseline disagreed that married HIV positive women would never use condoms (*n* = 32, 69.6%) and this increased further at endline (*n* = 37, 87.1%).

## Discussion

The success of this model in improving reproductive health and HIV service integration at healthcare service level has been demonstrated via provider feedback [36, 37]. Looking more specifically at client fertility desires, inferences can be made about the impact of the integrated service provision on HIV positive client family planning and fertility desires.

The proportion of PLHIV in this study who had desires for more children were low at both baseline and endline, demonstrating a need for integrated services that provide information on family planning methods to prevent unintended pregnancies in those HIV infected clients who do not want more children, as well as for services to facilitate safe and healthy pregnancies for those HIV infected clients who do want children [5].

Although reproductive desires and intentions may be adjusted, they are not necessarily changed by being HIV positive, and various factors could impact on those desires [20]. There has also been contradictory research, some demonstrating that knowledge of HIV-positive status has had little effect on pregnancy desires, and others that receipt of HIV-positive test results may lead to a significant reduction in pregnancy desires [21].

In our study, the most commonly reported issues affecting future fertility desires in PLHIV who both did and didn’t want more children, were concerns about the health of themselves and their child (ren), also reported in prior studies [20, 22–24]. Concerns have been related to poor state of health and fears that pregnancy could hasten HIV/AIDS progression [20, 24], as well as anxiety about the risks of HIV transmission to an infant [20, 22, 24] or partner [20]. Conversely, it has been shown that women with a positive health perception had a greater desire for more children [21]. The proportion of clients that were worried about their own health and the health of their children declined at endline in our study, which could be related to increased proportion on treatment and possibly decreased concerns about own health, as well as improved education and integration of services as a result of the integration model.

More specifically, different characteristics of HIV infected men and women may influence desires for more children, and patterns of these desires are evident in our data. In terms of relationships, HIV infected men and women in stable relationships at both baseline and endline in our study were more likely to desire more children. Although reasons for this were not explored, other research has also found that being in a main partner relationship (with fewer children) was significantly associated with fertility desires [41]. Desires not to have children have been outweighed by partner and family expectations/decisions to have more children [20, 23], and cultural expectations to prove fertility in long term relationships [24], indicating that women in more stable relationships may be following their partner desires. With respect to number of existing children, our study mirrored other research, whereby at both baseline and endline, HIV infected women with fewer children were more likely to want more children [21, 41]. Our study did not demonstrate any patterns in HIV infected men’s number of existing children and future desire for children.

Partner HIV status has also been linked to future desires to have children [3]. Although, in our study, the number of HIV positive female clients who wanted more children and had HIV positive male partners, was low across both baseline and endline, there was a marginal increase in numbers at endline. This could possibly be linked to improved integration, where women are educated about the different options for having children safely with HIV infected partners. Although our model did not specifically include a module on safer conception in HIV infected couples, the focus on integration of FP and HIV across services, as well as improved referral systems, could have meant that clients were more exposed to safer conception messages. There were no clear patterns in our study of HIV positive male clients’ desires for children, whether they had HIV positive or HIV negative partners.

HIV-positive status and desires for children may also be affected by availability of HIV treatment and PMTCT services, and desires to have children may vary when comparing women on ART, with those women who are from the pre-ART era [3, 25]. A study in Vietnam found that women on ART were twice as likely as those not on treatment to want a child/another child in future [13]. If and when people have access to treatment, concerns about their own health and health of their babies may be fewer [3, 20]. Furthermore, people on treatment have more regular access to the health system, and may therefore have increased awareness about how to have children safely. In our study, the proportion of people wanting more children who were on antiretroviral treatment, increased between baseline and endline. Proportions of people accessing ART would have increased over this time, but healthcare providers in these facilities received training on ART and FP, as well as referral and management of HIV clients, as part of our integration model, so would have been able to provide PLHIV with more support for having children safely.

Contraceptive uptake of PLHIV is impacted by lack of information on available and/or suitable methods [7], as well as concerns of side effects and interactions with antiretroviral treatment [24]. In our study, similar to other research [13], despite not having future fertility desires, some HIV infected women did not use a method to prevent pregnancy. However, this behavior decreased at endline. Improved uptake of contraception at endline within these facilities could be linked to improved HIV and FP service integration offered as a result of our model. More specifically healthcare provider and health systems navigator training on how to integrate HIV and FP services, as well as on the importance of dual protection, and ARV and contraceptive method interactions, could have resulted in improved education and support for contraceptive use in HIV infected people at these facilities.

Healthcare providers’ attitudes shape reproductive choices available to women [20, 34], and therefore are important influencers in fertility decision making. Researchers have documented negative provider attitudes to HIV positive women who want to be pregnant [42], and some have noted that providers have advised HIV infected women to abstain from sex [13]. Healthcare providers in our study were largely supportive of PLHIV having children, however, there were some at baseline and endline who did not think that PLHIV should have children. One way to address negative provider attitudes is to provide training in multiple service delivery points, which should focus on assessing fertility desires and on referral to family planning and safe pregnancy services [17], as well as on accurate information on safer conception practices. There is also a clear need to destigmatise issues around HIV and childbearing in the public health sector [43].

Our data revealed that healthcare provider views became more supportive and less judgmental by endline. These supportive attitudes could be related to the improved training and support that healthcare providers across the different service delivery points received – in referral and values clarification - as part of the integration model.

Whether PLHIV want more children or not, they are conceiving and therefore need access to quality information on family planning and contraceptive options. It has been recommended that in order to enable HIV infected women to avoid unintended pregnancies, efforts must be made to provide information and access to contraceptive services, and linkages to HIV care and treatment services access must be strengthened [20]. Through our integration model, we were able to provide healthcare providers with support and education which facilitated integration of SRH services. In addition, health systems navigators were able to facilitate community level education and training to improve community access to information and services. In future, provider education and training should focus on the various factors which have been identified as influencers in future desires for children. By including these in training, provider messages can be more focused on the specific needs of the clients that present to them with reproductive needs. In this way, the healthcare providers can provide appropriate integrated reproductive health services for PLHIV.

### Limitations

There are limitations to this study. It was conducted some time ago, and since then there have been changes in the healthcare environment - the HIV treatment environment has changed, provider capacity has improved due to increased focus on integration, and the time lapsed since the implementation of safer conception guidelines means that it is possible that they are now being implemented in a more appropriate manner. However, numerous challenges to service integration still exist, and guideline implementation and uptake of family planning continues to be a challenge [8, 30, 44]. In addition, there is little previous data on fertility desires of men living with HIV, and factors influencing these, therefore this data is novel and still bears relevance, and can be used to inform healthcare provider training.

The integration model was developed and tested over a 3 year period. The model was not monitored after the study was completed, but it is hoped that the integration practices learnt continued in the study setting once the study ended. Health systems navigators are now used in other South African service delivery settings [45].

The baseline/endline cross-sectional design did not allow for us to determine the impact of the context on the findings. Although some of our study findings point to the success of the integration model, considerations such as improved integration guidelines and increased uptake of ART in the health sector could have impacted on study findings. However, these issues and possible influencers have been further described in the discussion.

Furthermore, there were discrepancies in proportions of clients attending different clinics in baseline and endline. This is due to a number of factors, including more clients accessing more than one service at their visit at endline compared to the baseline visits, and also due to the client load for the different services during the days of interviewing. There were higher numbers of participants recruited at particular services at endline, to include sufficient male clients in the study.

The sample size of healthcare providers was small, but it is representative of providers working at the healthcare facilities selected by the Department of Health. High workload and turnover of healthcare providers in healthcare facilities meant that it was not possible to sample the same providers at both baseline and endline.

In addition, due to the nature of survey data, reasons for wanting/not wanting more children were not explored in relation to the various influencing factors. However, parallels have been made with other literature in order to interpret possible influencers.

## Conclusions

Many HIV positive men and women are concerned about their own health and that of any children that they may have in the future, with many reporting that they do not want any more children. Those who are considering having children need counseling and support in pregnancy. Various characteristics of PLHIV may affect their fertility desires. Healthcare provider training should focus on factors influencing desire for children. In addition, the success of this model demonstrates a need for continued focus on integration of family planning and reproductive choices with HIV services. Finally, healthcare provider training on policies and programs to address the reproductive health desires of HIV positive men and women is required.

## Supplementary Information

**Additional file 1.** Client baseline questionnaire. Client endline questionnaire. Provider baseline questionnaire. Provider endline questionnaire.

**Additional file 2: Figure 1.** Model diagram.

## Data Availability

The datasets generated and/or analysed during this study are not publicly available due to participant confidentiality and ethics limitations. However, they may be available from the corresponding author upon reasonable request and on submission of proposal which will need approval by the relevant ethics committee. The surveys conducted were developed specifically for this study, and have been included as supplementary files to this submission.

## Abbreviations

ANC: Antenatal clinic
ART: Antiretroviral therapy
ARV: Antiretrovirals
BREC: Biomedical Research Ethics Committee
DoH: Department of Health
FP: Family planning
HAART: Highly active antiretroviral therapy
HCT: HIV counselling and testing
HREC: Human Research Ethics Committee
PHC: Primary healthcare
PLHIV: People living with HIV
PMTCT: Perinatal mother-to-child transmission
SRH: Sexual and reproductive health
STI: Sexually transmitted infection
TOP: Termination of pregnancy
