# Supplementary material for: Fertility desires of people living with HIV: does the implementation of a sexual and reproductive health and HIV integration model change healthcare providers’ attitudes and clients’ desires?
Source: BMC Health Serv Res. 2021 May 26;21:509. doi: 10.1186/s12913-021-06487-0 (PMC8157636; doi:10.1186/s12913-021-06487-0)
Supplement: Supplementary file 1 — Additional file 1. Client baseline questionnaire. Client endline questionnaire. Provider baseline questionnaire. Provider endline questionnaire. [file 12913_2021_6487_MOESM1_ESM.zip › Additional file 2_Client Endline Questionnaire (25 July 11)R4.pdf]

Integration: Client Endline Assessment

INSTRUCTIONS TO INTERVIEWER: Explain the project to the participant. If he/she agrees to participate, obtain written consent by requesting him/her to sign on the space provided on the consent form provided before proceeding.

Note: For each of the questions listed below, circle the code that best represents the clients' response, or write the client's own words (verbatim) for the open ended questions.

Facility Name/Section:<br>Igama lesikhungo/isigaba:	
Type of facility:<br>Uhlobo lwesikhungo:	1= Hospital 2= Primary health care centre<br>3= Clinic 77= Other (specify)_________
Service:<br>Usizo(benefit):<br><br>*[more than one may be circled if necessary]*	1= PHC 2= FP<br>3= ANC 1st visit 4= ANC follow-up<br>5= STI 6= HCT<br>7= HIV/ART 8= Immunization/post-natal<br>77= Other (specify)____________________________
Client ID:	
Interviewer code:	
Date of interview (Date/month/year):	___ ___/___ ___/ ________<br>dd mm yy
Interview start time:	
Interview stop time:	
Results codes:	1= Complete 2= Partly complete<br>3= Refused to continue

CHECKED BY: …………………..……………………………………………..

DATE CHECKED: …………………………………………………………...

DATA ENTRY BY: ……………………………………………………………..

DATA ENTRY DATE: ………………………………………………………….

1. ALL CLIENTS

The following section is to be completed for all clients being interviewed:

	Question	Response	Skip
1.1	How old are you?	Age in completed years _________	
1.2	Gender?	Male…..1<br>Female…..2	
1.3	What is your highest level of education completed?	None …..1<br>Primary level…..2<br>Secondary level…..3<br>Matric…..4<br>Tertiary…..5	
1.4	I would now like to ask you about your present relationship or relationships? Are you currently:<br><br>*[Interviewer to read responses and circle all that apply]*	Married – living together…..1<br>Married – living apart…..2<br>Not married, living with partner…..3<br>Regular visiting partner…..4<br>Single, no current partner…..5<br>Multiple/concurrent partners…..6<br>Other (specify)____________….77	
1.5	Do you have one primary sexual partner or do you have any casual partners?<br><br>*[Read responses and circle one]*	One regular/primary partner…..1<br>Casual partners only…..2<br>Regular partner and casual partners…..3<br>None, single…..4	
1.6	What is your religion?	Christian…..1<br>Catholic…2<br>Muslim…..3<br>Hindu…..4<br>Atheist/Agnostic…..5<br>Ancestral worship…..6<br>Other (specify)____________….77	
1.7	Are you currently working?<br><br>*[employment is any job that earns you money]*	Yes, employed full/part time/self employed…..1<br>No, unemployed…..2<br>Student/scholar…..3<br>Housewife…..4<br>Other (specify)____________....77	

1.8	How many children do you currently have?	None…..1<br>One…..2<br>Two…..3<br>Three…..4<br>Four or more…..5	
1.9	Do you want to have more children?	Yes…..1<br>No…..2<br>Not sure…..3	
1.10	Was your/your partner's last pregnancy planned?	Yes…..1<br>No…..2<br>Don't know…..88	
1.11	What time did you arrive at the clinic/hospital today?		
1.12	How long did you wait to see the health care provider?	Time waited:_________________	If <1 hour, go to 1.14
1.13	**If an hour or more**, how much did it bother you having to wait?	Bothered me a lot…..1<br>Bothered me a bit…..2<br>I don't mind at all…..3	
1.14	Just to confirm, what was/were the reason/s for your visit today?<br><br>*[circle all that apply]*	PHC…..1<br>FP…..2<br>ANC 1st visit…..3<br>ANC follow-up…..4<br>STI…..5<br>HCT…..6<br>HIV care/ART…..7<br>Immunization/post-natal…..8<br>Other (specify)____________….77	

1.15	What other services have you used in this facility over the past year?<br><br>*[Circle all that client mentions]:*	PHC…..1<br>FP…..2<br>ANC 1st visit…..3<br>ANC follow-up…..4<br>STI…..5<br>HCT…..6<br>HIV care/ART…..7<br>Immunization/post-natal…..8<br>Other (specify)____________….77	
1.16	How do you normally reach the clinic?	Public transport, e.g. bus …..1<br>Mini bus taxi…..2<br>Metered taxi…..3<br>I walk…..4<br>Car…..5<br>Other (specify)____________….77	
1.17	How long does it normally take you to get to the clinic?	Time taken:__________________	
1.18	Did you feel like your consultation with the health care provider today was confidential and private (i.e. not overheard/seen by other clients)?	Yes, all of it……1<br>Some, but not all…..2<br>No…..3	
1.19	What do you think about the health providers of this health facility/department?<br><br>*[Do not read, circle all given answers]*	Good/supportive…..1<br>Caring…..2<br>Short tempered…..3<br>Not listening to clients…...4<br>Shouting…..5<br>Not caring…..6<br>Other (specify)________________<br>____________________________<br>____________________________<br>____________________________<br>____________________________<br>________________________….77	

1.20	Are all the services available all the time here, or do you need to come on different days for different services?	They are available on same day…..1<br>Need to come on different days…..2<br>Don't know/unsure....88	
1.21	In the last year, have you needed or received more than one service on one day?	Yes ….1<br>No…..2	If no, go to 1.24
1.22	**If yes**, please give your most recent example:	Service 1___________________<br>Service 2___________________<br>Service 3___________________<br>Service 4___________________	
1.23	Did you actually get these services on the same day?	Yes…..1<br>No…..2	
1.24	In the last year, have you come to the clinic for a service and been asked to come back on the next/different day?	Yes…..1<br>No…..2	
1.25	Have you ever had to queue more than once in one day for separate services?	Yes…..1<br>No…..2	
1.26	Do you think it is possible to get two services on the same day at this facility?	Yes…..1<br>No…..2	

1.27	If you have come to the clinic more than once this year did you see the provider you saw today on previous occasions?	Yes, same provider…..1<br>No, different provider…..2<br>Mostly the same provider……3<br>Have never been before/only been once this year…..4	
1.28	If given the choice, would you like to see the same provider each time you come to the clinic?	Yes…..1<br>No ...2	
1.29	During your consultation today, were you referred to any other provider for other services?	Yes…..1<br>No…..2	If no, go to 1.33
1.30	**If yes,** was this referral to another department in this facility or to another facility?	Another dept/service in this facility…..1<br>Another facility…..2	
1.31	Please name the place and for what service:<br><br>*[Note: ANC clients will normally see more than one provider on same day, but list their response anyway]*	Service_____________________<br><br>Place______________________	If place external to facility, go to 1.33

1.32	**If referral in same clinic only**:<br>Did you receive this additional service today, or do you have to come back on a different day?	Different provider, same day…..1<br>Told to come back another day…..2	
1.33	If you need two services (e.g. PHC/FP), would you prefer to have them on the same day in the same consultation?	Same day/ consultation…..1<br>Same day/different consultation…..2<br>Don't mind different day…..3<br>Other (specify)____________….77	
1.34	Are you happy with the way health services are in this facility/clinic or do you have any suggestions as to what might improve the way services are delivered for you and your family?<br><br>*[Do not read answers, circle all that apply]*	I am happy with services…..1<br>I would like to wait less…..2<br>I would like clinic to be open after hours…..3<br>Other (specify)________________<br>______________________________<br>______________________________<br>________________________....77	
[Read clearly]<br>Now I am gong to ask you some questions about family planning and sexual behavior. Some of the questions may be sensitive but remember, our conversation will be confidential.			
1.35	Do you currently use a method to prevent pregnancy yourself/with your partner?	Yes…..1<br>No…..2<br>Not sexually active/no partner…..3<br>Currently pregnant so not applicable…..4	If no, not sexually active or not applicable, go to 1.37

1.36	**If yes**, what method are you/your partner currently using?<br><br>*[Circle all that client mentions]:*	2-month injectable (NET-EN)…..1<br>3-month injectable (Depo)…..2<br>Combined oral contraceptive pills…..3<br>Progestin-only contraceptive pills (POPs)…..4<br>IUD…..5<br>Male condoms…..6<br>Female condoms…..7<br>Withdrawal…..8<br>Sterilization…..9<br>Emergency contraception….10<br>Abstinence….11<br>LAM (breastfeeding)….12<br>Fertility awareness-based/calendar-based Method/NFP….13<br>Thigh sex….14<br>Other (specify)_____________....77	
1.37	Which family planning methods do you think are available at this clinic?<br><br>*[Circle all that client mentions]*	2-month injectable (NET-EN)…..1<br>3-month injectable (Depo)…..2<br>Combined oral contraceptive pills…..3<br>Progestin-only contraceptive pills (POPs)…..4<br>IUD…..5<br>Male condoms…..6<br>Female condoms…..7<br>Emergency contraception…..8<br>Sterilization…9<br>Other (specify)____________.....77	
1.38	Where would a woman living in your area go if she wanted a termination of pregnancy?<br><br>*[Ask her/him to name actual facility]*	Don't know…..88<br>King Edward hospital…..1<br>Prince Mshiyeni hospital…..2<br>Wentworth hospital…..3<br>Other (specify)_____________....77	

1.39	Do you currently do anything to prevent sexually transmitted infections, including HIV?	Yes…..1<br>No….2<br>Not sexually active…..3<br>Don't know…..88	If no, D/K or not sexually active, go to 1.41
1.40	**If yes,** what method do you use?<br><br>*[Circle all that client mentions]:*	Male condoms…..1<br>Female condoms…..2<br>Abstinence…..3<br>Thigh sex…..4<br>Monogamy/One partner…..5<br>Partner reduction…..6<br>Male circumcision…..7<br>Other (specify)_____________....77	
1.41	If you use male /female condoms, how often do you use them?	Never use them…..1<br>< 50% (less than half the time)…..2<br>About 50% (half the time)…..3<br>>50% (more than half the time but not every time)…..4<br>100% (always)…..5	If don't use them, go to 1.46
1.42	Where do you get male condoms from?	Clinic…..1<br>Pharmacy…..2<br>Shop……3<br>Friend…..4<br>Other (specify): ____________....77	
1.43	**If you use condoms**, do you use condoms just for preventing sexually transmitted infections including HIV or do you also use them for preventing pregnancy?	For preventing STIs/HIV only…..1<br>For preventing pregnancy only…..2<br>For preventing STIs/HIV and pregnancy…..3<br>Other (specify)_____________...77	
1.44	Did you use a male/female condom during your last sexual intercourse?	Yes…..1<br>No…..2	If yes, go to 1.46

1.45	**If no**, why did you not use a male/female condom?	Had none available…..1<br>Partner would not wear one…..2<br>Want to get pregnant…..3<br>Partner and I never use condoms…..4<br>I don't use condoms with that partner…..5<br>Other (specify): ____________....77	
1.46	Have you ever heard of a female condom?	Yes…..1<br>No…..2	If no, go to 1.49
1.47	**If yes,** have you ever tried using one?	Yes…..1<br>No…..2	If no, go to 1.49
1.48	**If yes,** where did you get the female condom from?	Clinic…..1<br>Pharmacy…..2<br>Friend…..3<br>Other (specify): ___________ ….77	
1.49	**If no,** do you think you would ever use a female condom?	Yes…..1<br>No…..2<br>Not sure…..3<br>Never seen/don't know about them…..4	
1.50	Did you get any male/female condoms during your visit today?	Yes, from provider…..1<br>Yes, from dispenser…..2<br>No…..3	
1.51	Do you know anything about male circumcision?	Yes…..1<br>No…..2	If no, go to 1.54

1.52	**If yes,** can you tell me what male circumcision is?		
1.53	Where would you go if you/your partner wanted to be circumcised?	Wentworth Hospital……1<br>Traditional healer….2<br>Another facility<br>(Specify)________________…..77	
1.54	Have you heard anything about the potential for male circumcision to prevent STIs, including HIV?	Yes…..1<br>No…..2	If no, go to 1.56
1.55	**If yes,** what have you heard?	Reduces the risk of STI infection…..1<br>Reduces the risk of AIDS/HIV infection…..2<br>Improves hygiene…..3<br>Improves sex for the man…..4<br>Improves sex for women…..5<br>Other (specify)_________________<br>____________________________<br>____________________________<br>____________________________<br>________________________....77	
1.56	Have you ever heard of an IUD/Loop?	Yes…..1<br>No…..2	If no, go to 1.58

1.57	**If yes,** what have you heard about it?<br><br>*[Circle all that apply]*	A method for women that have had children…..1<br>A method for older women…..2<br>Don't know anything about it…..3<br>Can cause infections…..4<br>Very effective….5<br>Long-term method….6<br>Other (specify)________________<br>____________________________<br>____________________________<br>________________________....77	
1.58	Have you ever heard of emergency contraception/ morning after pill?	Yes…..1<br>No…..2	If no, go to introduction before q.1.61
1.59	**If yes**, where would you go if you wanted to get it?	Clinic…..1<br>Hospital…..2<br>Pharmacy…..3<br>Friend…..4<br>Other (specify): ____________....77	
1.60	**If yes,** have you/your partner ever used it before?	Yes…..1<br>No…..2<br>Not sure about whether partner has used it…..3	
[Read clearly]<br>Now I am gong to ask you some questions about HIV and HIV testing. Some of the questions may be sensitive but remember, our conversation will be confidential and you may refuse to answer any questions if you wish.			
1.61	Were you offered an HIV test today?	Yes….1<br>No….2	

1.62	Have you ever had an HIV test, today or any other time?	Yes…..1<br>No…..2	If yes, go to 1.64
1.63	**If not ever had test,** please can you tell me the reasons you decided not to take the test?<br><br>*[Circle all that apply]*	Scared of getting positive results…..1<br>I already tested negative before…..2<br>My partner did not want me to take the test…..3<br>Do not want to know my status…..4<br>There is nothing that can be done about HIV…..5<br>I know/ suspect that I am HIV+…..6<br>I am worried about confidentiality…..7<br>No-one has ever asked me to test…..8<br>I know I am not at risk from HIV…9<br>Other (specify)____________….77<br>Do not know….88	All to go to 1.73
1.64	**If client has had an HIV test:** Were you tested today, or when was the last time you were tested?<br><br>*Write 'today' if client tested today*	Insert date<br>___________	
1.65	How many times have you been tested before that?<br><br>*Write 'none' if first test*	Write number of times<br>___________	
1.66	Where did you get tested?<br><br>*[if client has tested more than once, circle all mentionedl]*	ANC/PMTCT this clinic…..1<br>ANC/PMTCT other facility.….2<br>HCT this clinic.….3<br>HCT other facility…..4<br>NGO….5<br>Private……6	
1.67	Why did you take the test?	Provider suggested test (eg ANC)…..1<br>Private insurance.….2<br>I asked for test…..3<br>Other (specify)_____________....77	
1.68	Have you received your results of your test?	Yes…..1<br>No, did not want to know…..2	If no, go to 1.75

		No, they did not give them to me 3<br>Did not receive them yet…4	
1.69	I would like to ask you if you would be prepared to share the results of your HIV test with me. This information will be kept confidential (I will not tell anybody else) and there is no name on this questionnaire. If you do not want to tell me that's no problem we can skip this question.	Positive…..1<br>Negative…..2<br>Did not wish to disclose…..3	If negative, or does not want to disclose, go to 1.71
1.70	**If positive,** have you had a CD4 blood test taken? This is a test that tells you if you need treatment?	Yes…..1<br>No…..2<br>Don't know/not heard of this…..3	
1.71	**If you know your HIV status and are in a relationship:** have you disclosed your HIV status to your partner?	Yes…..1<br>No…2	If yes, go to 1.74

1.72	Are you planning to tell your partner your status?	Yes…1<br>Maybe…2<br>No…3	If yes, go to 1.74
1.73	Why not?	My partner will leave me…1<br>My partner will beat me…2<br>I'm ashamed…3<br>I didn't get HIV from my current partner…4<br>I don't know how to tell him/her…5<br>Other (specify):________________<br>__________________________...77	
1.74	Did the provider ask you to encourage your partner to come for testing?	Yes…..1<br>No…..2	
1.75	Has your partner been tested for HIV?	Yes…1<br>No…2<br>Don't know…88	If no or don't know: if male go to 1.78, or if female go to next section
1.76	Do you know your partner's HIV status?	Yes…1<br>No…2	If no: if male go to 1.78, or if female go to next section
1.77	Would you be prepared to tell me your partner's status if you know it?	HIV Positive…1<br>HIV Negative…2<br>Not prepared to disclose…..3	

	**Male Clients:** *For the following questions, ask men clients only*		All women clients to go to next section
1.78	Have you ever come to the clinic with your partner for any services?	Yes…..1<br>No…..2	
1.79	**If yes**, which one?	HCT…..1<br>FP visit…..2<br>ANC Visit…..3<br>STI visit…..4<br>PHC visit…..5<br>Baby/child Visit…..6<br>Other (Specify)____________....77	
1.80	If you had a sexually transmitted infection, where would you go for treatment/advice?	This facility…..1<br>Another facility…..2<br>Hospital…..3<br>Pharmacy/chemist…..4<br>Traditional healer…..5<br>Other (Specify)____________....77<br>Don't know….88	

The following sections should only be asked if the client is attending a particular service.

2. ANTENATAL CLINIC

This section must be asked only to pregnant women who are coming for antenatal care visits. Only ask women who have had at LEAST two ANC visits previously.

	Question	Response	Skip
I would like to ask you personal questions about Antenatal care and the PMTCT program. I would like to assure you again that what ever we are going to discuss will remain confidential.			
2.1	Did you start ANC at this facility or elsewhere?	This clinic….1<br>Elsewhere (specify)__________...2	
2.2	Did you have a test for syphilis during your antenatal care?	Yes…..1<br>No…..2<br>Don't know….88	
2.3	Have you been offered an HIV test during this pregnancy?	Yes…1<br>No…2<br>Don't know…88	
2.4	**If client HIV positive (from Q.1.69 above):**<br>Are you enrolled in or will you join the PMTCT programme?<br>*[explain PMTCT programme if client does not know: See below]*	Yes…1<br>No…2<br>Don't know…88	
If doesn't understand PMTCT read clearly: *If a mother is HIV positive she can pass HIV to her baby during pregnancy, labour, childbirth and breastfeeding. To prevent mother-to-child transmission all pregnant women are offered HIV testing during their ANC. If she is HIV positive there are medicines that will be given to her and her baby to lower the chances of passing HIV to the baby. Because HIV can also be passed to the baby during breastfeeding the health providers will discuss with the mother how she can feed her baby so she can lower the chances of passing HIV to her baby while she is breastfeeding.*			
2.5	Will you abstain/are you abstaining from sexual intercourse during this pregnancy?	Yes…..1<br>No…..2<br>Undecided…..3	If no or undecided, go to 2.7
2.6	**If abstaining**, what month of your pregnancy did you start/will you start to abstain?<br>*[Write month 1-9]*	Month:__________________	

	Question	Response	Skip
2.7	Have you used condoms during this pregnancy?<br><br>*[Check if answer corresponds to answer in q1.44]*	Yes…..1<br>No…..2	

During this pregnancy did the providers talk to you about:		Yes	No	DK
2.8	HIV counseling and testing (HCT)	1	2	88
2.9	Prevention of mother to child transmission of HIV (PMTCT)	1	2	88
2.10	Medicine/pill that prevents spread of HIV to the new born baby (NVP/Nevirapine)	1	2	88
2.11	Prevention of STI/HIV/AIDS	1	2	88
2.12	Importance of using condoms	1	2	88
2.13	Demonstrated how to use a condom	1	2	88
2.14	Family planning	1	2	88
2.15	Infant Feeding choices	1	2	88
2.16	Tuberculosis (TB)	1	2	88

3. POSTNATAL/WELL BABY CLINIC

This section must be asked only to women who have babies that are 12 months old or below, from the post natal clinic, well baby clinic mothers, or immunizations.

	Question	Response	Skip
3.1	Are you here for a postnatal visit, immunization, or visit for your baby?<br><br>*[Circle all that apply- check response for what was reason for visit today]*	Post natal visit…..1<br>Immunization…..2<br>Well baby…..3<br>Other (specify)_______________…77	
3.2	How old is your baby now?	Completed months _________	
3.3	Did you have a test for syphilis during your antenatal care?	Yes…..1<br>No…..2	

		Don't know…..3	
3.4	Were you offered an HIV test during your last pregnancy?	Yes…..1<br>No…..2	If not HIV positive (q1.69, section 1), go to 3.11
3.5	**If HIV positive (q 1.69, Section 1),** did you join the PMTCT program?<br><br>*[explain PMTCT programme if client does not know what it is: See Section 2.4]*	Yes…..1<br>No…..2	If yes go to 3.7
3.6	**If not joined,** why did you not join the programme?	Too busy/too many appointments…..1<br>Did not need to come…..2<br>Did not want people to know my status…..3<br>I did not know about the program…..4<br>Other (specify)______________....77<br>Don't know…88	All go to 3.11
3.7	**If yes**, are you still on the program?	Yes…..1<br>No…..2	If no go to 3.11
I would like to ask you personal **questions about HCT and the PMTCT program**. I would like to assure you again that whatever we are going to discuss will remain confidential.			
3.8	Have you been coming for your monthly appointments?	Yes, always…..0<br>Yes but not always…..1<br>No…..2	If yes always or yes but not always, go to 3.10
3.9	**If no,** why are you not coming for your appointments/not on the programme?	Too busy/too many appointments…..1<br>Did not need to come…..2<br>Did not want people to know my status…..3<br>Other (specify)______________....77	
3.10	If attending/attended PMTCT: What was/is done when you come/came for PMTCT program follow-up?<br><br>*[Read out all options and circle all that apply]*	Health education on baby feeding…..1<br>Immunization…..2<br>Weighing of the baby…..3<br>Infant feeding support to mothers…..4<br>Counselling…..5<br>Family planning…..6<br>Collecting milk powder…..7<br>Infant HIV test…..8<br>Other (specify)______________….77	

3.11	Where did you deliver this last baby?	King Edward VIII…..1<br>Prince Mshiyeni…..2<br>Wentworth…..3<br>Cato Manor clinic…..4<br>Addington…..5<br>R.K. Khan…..6<br>Mahatma Gandhi…..7<br>Clairwood…..8<br>Inkosi Albert Luthuli…..9<br>Home…..10<br>Other (specify)______________....77	
3.12	Did you *yourself* go to the clinic for a post-natal check up? This is a check up for yourself and not just for baby immunization/FP.<br><br>*[Do not ask if she is here for the postnatal check up].*	Yes…..1<br>No…..2	
3.13	Did you get a physical examination at your visit today?	Yes…..1<br>No…..2	
3.14	Did your baby get a physical examination at the visit today?	Yes…..1<br>No…..2<br>Baby not with mother today…..3	
3.15	Did you discuss family planning in today's visit?	Yes…..1<br>No…..2	
3.16	Did you abstain from sexual intercourse during this pregnancy?	Yes…..1<br>No…..2	
3.17	**If yes,** what month of your pregnancy did you start to abstain?<br><br>*[Write month 1-9]*	Month _____________________	
3.18	Did you use condoms in your last pregnancy?	Yes…..1<br>No…..2	

4. PHC SERVICES

This section must be asked to men or women who are attending for PHC services including STI.

	Question	Response
4.1	What was the reason you came to see the doctor or nurse today?<br><br>*[Circle all that apply]*	Broken bone/s…..1<br>Other injury (specify)______________________…..2<br>TB…..3<br>Cough/cold/flu…..4<br>Asthma/other lung problem…..5<br>Heart problems…..6<br>Nausea/vomiting/diarrhoea…..7<br>Other child illness (specify)________________…..8<br>Mental health concern…..9<br>Dentistry…..10<br>Ear infection/problem….11<br>RTI/STI….12<br>Eye problem….13<br>Joint problem/arthritis….14<br>Prescription refill….15<br>FP….16<br>Other (specify)___________________________...77
4.2	Who did you see at the clinic today?<br><br>*[Circle all that apply]*	Doctor…..1<br>Nurse……2<br>Pharmacist….3<br>Counsellor…..4<br>Other (specify)_______________...77

5. FAMILY PLANNING/UKUHLELA UMNDENI?

This section must be asked to women who have come for family planning.

	Question	Response			Skip
5.1	Is this your first time visiting this clinic for family planning or is this a repeat visit?	First time…..1<br>Repeat …..2			If first time, go to 5.3
5.2	***Returning client:*** When was your last visit?				
First time clients: During today's family planning visit did the providers offer you:-<br><br>*Repeat clients:* In any of the last year's family planning visits did the providers offer you:-					
		**Yes**	**No**	**D/K**	
5.3	HIV counseling and testing (HCT)	1	2	88	
5.4	Pelvic/ Genital examination	1	2	88	
5.5	Pap smear	1	2	88	
5.6	Demonstration on how to use a male condom	1	2	88	
5.7	Demonstration on how to use a female condom	1	2	88	
First time clients: During today's family planning visit did the providers talk about:-<br><br>*Repeat clients:* In the last year's family planning visits did the providers talk about:-					
5.8	HCT	1	2	88	
5.9	Male condoms	1	2	88	
5.10	Female condoms	1	2	88	
5.11	Dual method use (condoms plus another method)	1	2	88	
5.12	Tuberculosis (TB)	1	2	88	
5.13	Cervical or breast cancer	1	2	88	
5.14	CTOP	1	2	88	
5.15	**For repeat clients:**<br>Did you switch methods today?	Yes…1<br>No…2			If did not switch, go

	Question	Response	Skip
			to 5.17
5.16	**For new clients, or those who switched methods:** What methods of contraception were discussed with you today?<br><br>*[Circle all that client mentions]:*	Injectable contraceptives…..1<br>Combined oral contraceptives (COCs)…..2<br>Progestin-only oral contraceptives (POPs)…..3<br>Male condoms…..4<br>Female condoms…..5<br>Intrauterine devices (IUDs)…..6<br>Emergency contraception…..7<br>Sterilisation…..8<br>Other (specify):_________....77	
5.17	Did you get given any family planning method from the nurse today?	Yes…..1<br>No…..2	If no, go to next section
5.18	**If yes**, did you get ________________ method?<br><br>*[the one client said s/he was using earlier in the questionnaire, question 1.36]*	Yes…..1<br>Did not get method today…..2<br>Other (specify):_________....77	

6. HCT

This section must be asked to clients who have had HCT today (including people who had HCT within another consultation).

	Question	Response	Skip
6.1	Did you sign an informed consent to get the HIV test?	Yes…..1<br>No…..2	

During this visit, did the providers talk to you about:				
		**Yes**	**No**	**D/K**
6.2	Risk reduction, i.e. reducing the risk of getting STIs/HIV?	1	2	88
6.3	Monogamy i.e. Importance of being faithful to one partner?	1	2	88
6.4	Safer sex	1	2	88
6.5	PMTCT	1	2	88
6.6	How to tell your HIV status to others/partner	1	2	88
6.7	Talking to your partner about also being tested	1	2	88
6.8	Treatment options for those who have HIV	1	2	88
6.9	The need to come back again for a repeat test	1	2	88
6.10	Tuberculosis (TB)	1	2	88
6.11	STIs	1	2	88
6.12	Importance of family planning for people who have HIV	1	2	88
6.13	Domestic violence	1	2	88
6.14	Prevention of STI/HIV/AIDS	1	2	88
6.15	Importance of using condoms	1	2	88
6.16	Demonstrated how to use a condom	1	2	88
6.17	How to negotiate or talk about condom use with your partner?	1	2	88
6.18	Dual method use, that is using both condoms and another contraceptive method at the same time	1	2	88

7. HIV/ARV SERVICES

This section must be asked to clients coming in for HIV/ARV services or wellness. PHC clients that respond to having HIV test and are positive can continue this section.

	Question	Response	Skip
7.1	Which service(s) did you come to the clinic for today?<br><br>*If client has completed another section and is positive ask:*<br><br>What HIV services have you used?	Wellness…..1<br>Treatment literacy classes…..2<br>ARVs…..3<br>Not used any HIV services yet....4<br>Other _____________ (specify)…77	If not wellness, go to q.7.3

7.2	If you came in for wellness, what issues did the nurse/doctor discuss with you?<br><br>*[Read out all options and circle all that apply]*	Safer sex…..1<br>STIs…..2<br>Treating opportunistic infections (e.g. TB, pneumonia,meningitis)…..3<br>Signs of deteriorating health…..4<br>When to return for CD4 blood count…..5<br>Getting sexual partners/children tested…..6<br>Nutrition…..7<br>Treatment options…..8<br>Tuberculosis…..9<br>PMTCT….10<br>Family planning….11<br>Emergency contraception….12<br>CTOP….13<br>Possible violence on disclosure….14<br>Other (specify)_____________....77	
7.3	Do you understand what a CD4 count is?	Yes…..1<br>No…..2	If no, go to 7.5
7.4	**If yes**, please explain:		
7.5	Did the provider ever ask you about your children or partner getting an HIV test?	Yes…..1<br>No…..2	
7.6	Did the provider talk to you about condom use?	Yes…..1<br>No…..2	

7.7	*Check questions 1.35 & 1.36.* ***If using contraception****, ask:*<br><br>Where do you get your method from?	This HIV/AIDS/wellness clinic…..1<br>FP service in this facility…..2<br>From another facility…..3<br>Other (specify)______________....77	If HIV/AIDS/ wellness clinic or other, go to 7.9
7.8	**If you get contraception from FP service in this or another clinic,** does the FP service you normally go to know you are HIV positive?	Yes, I told them…..1<br>Yes they asked…..2<br>No, they don't know…..3<br>I don't know if they know my status…..4	
7.9	Has your provider ever mentioned a support group in this clinic/area?	Yes…..1<br>No…..2	If no, go to 7.11
7.10	**If yes**, have you ever gone to a meeting?	Yes, in this facility…..1<br>Yes, outside this facility……2<br>No……2	If yes, go to 7.12
7.11	**If no**, or not in this facility would you attend a support group if the facility had one?	Yes…..1<br>No…..2	
7.12	Are you currently taking ARVs?	Yes…..1<br>No…..2	If no, go to next section
7.13	**If yes**, how long have you been taking ARVs?	[ ] [ ]<br>Years Months	

7.14	Where did you initiate your treatment regimen?	Wentworth Hospital…..1<br>Prince Myesheni Hospital…..2<br>Private doctor…..3<br>Other (specify): _____________....77	
7.15	Would you prefer to be able to pick up your monthly ARVs at the local clinic or at the hospital?	Local clinic…..1<br>Hospital…..2	
7.16	Please explain your choice.	Clinic would be more convenient……1<br>Prefer hospital- more confidential…..2<br>Other (Specify)______________….77	

8. FUTURE PREGNANCIES

Ask HIV positive men and women only (see q 1.68)

	Question	Response	Skip
8.1	Were you given any family planning advice when your status was confirmed or when you started ARVs?	Yes, at both points…..1<br>Yes, when status confirmed only…..2<br>Yes, when started ARVs only…..3<br>No…..4	If no, go to 8.3 or 8.4
8.2	What advice was given?<br><br>*[Circle all that apply]*	Advised to use contraception (state which method______________)…..1<br>Advised not to get pregnant…..2<br>Advised not to get pregnant until CD4 count increased…..3<br>Advised to seek counseling before getting pregnant…..4<br>Advised about risks of getting pregnant…..5<br>Advised that it is ok to have children once ARV treatment started…..6<br>Advised that it is ok to have children with management of disease…..7<br>Advised to stop having sex…..8<br>Other (specify)______________….77	
8.3	***If HIV positive and wants more children (see q.1.9):***<br>You said earlier in the Questionnaire that you would want more children (q 1.9). Has your HIV status affected your decision to have more children?	I have been told not to have them…..1<br>I am worried they may get sick/die…..2<br>I am worried about my health…..3<br>I am waiting for my viral load to decrease…..4<br>It hasn't changed anything…..5	

	[Circle all that apply]	I am getting advice on the best time to try and fall pregnant…..6<br>Other (specify)________________<br>___________________________....77	
8.4	***If HIV positive and wants no more children (see q.19.):***<br>You said earlier in the Questionnaire that you would not want any more children (q 1.9). Is it your HIV status that has affected your decision to have no more children?	Yes…..1<br>No…..2<br>Unsure…..3	If no or unsure, go to next section
8.5	**If yes,** how has your HIV status affected your decision to have no more children?<br><br>*[Circle all that apply]*	I have been told not to have them…..1<br>I am worried they may get sick/ die…..2<br>I am worried about my health…..3<br>I have children already…..4<br>Other (specify)_____________....77	

9. VALUES, BELIEFS AND ATTITUDES, SATISFACTION

To ask all clients this section.

I would like to have your opinion on the following statements. Some of the questions may be sensitive but whatever information is obtained will remain confidential. I will show you a card: please choose which is the most accurate in terms of whether you agree or disagree with the statement.

	Read the given statement and the scale to the participant and circle the number on the scale she or he feels best describe how participant feels	**1= strongly agree**<br>**2= somewhat agree**<br>**3= not sure**<br>**4= somewhat disagree**<br>**5= strongly disagree**				
9.1	It is ok to have sex at any age provided you use protection	1	2	3	4	5
9.2	Nurses do not give women under 16 family planning services	1	2	3	4	5
9.3	It is hard to talk to health providers because they shout at clients	1	2	3	4	5
9.4	Communities are not doing enough to strengthen morality in order to prevent spread of HIV	1	2	3	4	5
9.5	The government is not doing enough stop people that are spreading HIV	1	2	3	4	5
9.6	The health providers are doing their best for people with STIs, including HIV	1	2	3	4	5
9.7	It is time for men to be involved in maternal care of their partners	1	2	3	4	5
9.8	It is no use trying to educate men on condom use because men will never use condoms	1	2	3	4	5
9.9	There is no need to have termination of pregnancy services in this community because we believe in saving lives	1	2	3	4	5

I would now like to have your opinion on the quality of the services that you received today. I am again going to read you a series of statements. I will show you a card: please choose which is the most accurate in terms of whether you agree or disagree with the statement.						
	Read the given statement and the scale to the participant and circle the number on the scale she or he feels best describe how participant feels	**1= strongly agree**<br><br>**2= somewhat agree**<br><br>**3= not sure**<br><br>**4= somewhat disagree**<br><br>**5= strongly disagree**				
9.10	I was greeted warmly today	1	2	3	4	5
9.11	Staff were friendly	1	2	3	4	5
9.12	The nurses/doctors were easy to understand	1	2	3	4	5
9.13	The nurses/doctors listened to me	1	2	3	4	5
9.14	Staff were helpful in providing information	1	2	3	4	5
9.15	I felt free to ask questions	1	2	3	4	5
9.16	I was provided all the information I wanted during today's consultation	1	2	3	4	5
9.17	My consultation was private	1	2	3	4	5
9.18	The nurses/doctors ensured me about confidentiality	1	2	3	4	5
9.19	The waiting time was reasonable	1	2	3	4	5
9.20	The staff treated me with respect	1	2	3	4	5
9.21	I would like to come back to this clinic/hospital again	1	2	3	4	5
9.22	I would recommend this clinic/hospital to a friend	1	2	3	4	5

10. HEALTH SYSTEMS NAVIGATORS (ONLY AT WENTWORTH, CHESTERVILLE AND AUSTERVILLE)

Recently health systems navigators (HSNs) started working in this facility. Their role is to assist clients, like yourself, to find different services that you are referred to.

	Question	Response	Skip
10.1	Had you heard about the HSN/s before your visit today?	Yes, heard from friend….1<br>Yes, heard from family….2<br>Yes, seen at previous facility visit….3<br>Yes, other source (specify)______________....4<br>No….5	
10.2	Have you approached/spoken to an HSN today or any other day whilst visiting this facility?	Yes, today only…..1<br>Yes, on another day…..2<br>Yes, on more than one occasion….3<br>No……4	If no, go to 10.6
10.3	During your last contact with an HSN, did the HSN assist you?	Yes…..1<br>No……2	If no go to 10.6
10.4	**If yes**, how did they assist you?<br><br>*[Circle all that apply]*	Health talks……1<br>Directions……..2<br>They escorted me to my referral service point…3<br>Other (specify)________________<br>___________________________....77	
10.5	How would you rate the usefulness of your interaction with the HSN?<br><br>*[Circle number]*	Very useful….1<br>Somewhat useful….2<br>Not sure….3<br>Not really useful….4<br>Useless….5	All to go to q 10.7
10.6	**If no**, why were you not assisted by the HSN?	I did not need assistance…1<br>The HSN was busy…2<br>Other (specify)________________<br>___________________________....77	
10.7	What are your thoughts or opinions about having HSNs working in this facility? Do you think it is a good or bad idea, or a helpful or unhelpful idea?		

10. 8	Have you heard anyone giving health talks either at this facility or in your community?	Yes…..1<br>No.…..2	If no end of interview.
10.9	**If yes,** where did you hear these talks?<br><br>*[Circle all that apply]*	In my community….1<br>In the facility (specify where)_______<br>____________________________….2<br>Other (specify)__________________<br>___________________________....77	
10.10	Do you know who gave the talk?	Yes…..1<br>No.…..2	If no go to 10.13
10.11	Was it a…<br><br>*[Circle all that apply]*	Health systems navigator (HSN)…1<br>Community Healthcare Worker (CHW)….2<br>NGO (specify)_________________<br>__________________________....3<br>Other (specify)_________________<br>___________________________....77	
10.12	What were the talks about?<br><br>*[Circle all that apply]*	FC demonstration.…1<br>Adherence….2<br>Family planning….3<br>STIs…4<br>HIV….5<br>Gender based violence…..6<br>Other (specify)___________________<br>_______________________________<br>_______________________________<br>_______________________________<br>_______________________________<br>__________________________ 77	
10.13	Were the talks useful?	Yes…..1<br>No.…..2<br>Some (Specify which)_____________<br>__________________________........3	
10.14	Were you given any material at any of these talks?	Yes…..1<br>No.…..2	
10.15	**If yes**, what material did you receive?	Pamphlets…1<br>Other (specify)___________________<br>___________________________....77	

10.16	Are these materials useful?	Yes…..1<br>No.…..2	
10.17	Did you/Do you plan to access any services at a health care facility because of information you received at these talks?	Yes…..1<br>No.…..2	If no, end here.
10.18	**If yes,** did you/will you access the service at this facility or another facility?	This facility…..1<br>Another facility (specify)___________<br>_____________________________....2	
10.19	What service was it?	PHC…..1<br>FP…..2<br>ANC 1st visit…..3<br>ANC follow-up…..4<br>STI…..5<br>HCT…..6<br>HIV care/ART…..7<br>Immunization/post-natal…..8<br>Other (specify)_______________….77	

This is the end of our interview. Thank you so much for sharing your ideas with me. Do you have any questions, or is there anything that you would like to add before we end? If you have further thoughts about any of the issues we discussed today, please call Prof Jenni Smit, the South African Principal Investigator of the study, whose details are on your consent form and information sheet.
