# Supplementary material for: Fertility desires of people living with HIV: does the implementation of a sexual and reproductive health and HIV integration model change healthcare providers’ attitudes and clients’ desires?
Source: BMC Health Serv Res. 2021 May 26;21:509. doi: 10.1186/s12913-021-06487-0 (PMC8157636; doi:10.1186/s12913-021-06487-0)
Supplement: Supplementary file 1 — Additional file 1. Client baseline questionnaire. Client endline questionnaire. Provider baseline questionnaire. Provider endline questionnaire. [file 12913_2021_6487_MOESM1_ESM.zip › Additional file 3_Provider Baseline Questionnaire (6 July09)R4.pdf]

Integration: Provider Baseline Assessment

Note:

For each of the questions listed below, circle the code that represents clients' response or write client's own words (verbatim) for open ended questions.

Facility Name/Section:		
Type of facility:	1= Hospital<br>3= Clinic	2= Community health centre<br>77= Other (specify) __________
Type of provider:	1= Registered nurse<br>3= Enrolled assistant<br>5= Medical doctor<br>77= Other (specify)____________________________	2= Enrolled nurse<br>4= Lay counselor<br>6= Advanced midwife
Provider identification number:		
Interviewer code:		
Date data collected (Date/month/year):	______/___________/ ________	
Interview start time:		
Interview stop time:		
Results codes:	1= Complete<br>3= Refused to continue	2= Partly complete

CHECKED BY: ……………………………………………………………………..

DATE CHECKED: …………………………………………………………………...

DATA ENTRY BY: …………………………………………………………………..

DATA ENTRY DATE: ………………………………………………………………….

1. BACKGROUND INFORMATION

I am going to ask you some questions about yourself.

	Question	Response
1.1	How old are you?	Completed years ______
1.2	Gender	Female…..1<br>Male 2
1.3	What is your highest academic qualification?	Diploma…..1<br>Degree…..2<br>Certificate…..3<br>Other (specify)_________________….77
1.4	How many years have you been working as a health provider, including your training?<br>*[Duration of experience in the health profession including training]*	Number of years:________________
1.5	Which department/service are you working in at the moment?<br>*[Circle all given answers]*	Antenatal clinic….. 1<br>PMTCT…..2<br>Post natal…..3<br>Family Planning…..4<br>STI…..5<br>ARV…..6<br>VCT…..7<br>PHC (non-specific)…..8<br>CTOP…..9<br>Other (specify)_________________….77
1.6	What is your duration of work in the present department/service? *[Call it by name and round up to the nearest year]*	Years ……………
1.7	Have you ever considered taking a post outside the public health sector?<br>*[Circle all that apply]*	Yes overseas…..1<br>Yes private sector…..2<br>Yes municipality…..3<br>Never …..4<br>Other (specify) _____________________<br>__________________________________<br>_____________________________ ...77

1.8	Do you think the recent pay increase for nurses will encourage nurses to stay in public sector healthcare?	Yes ….1<br>No….. 2<br>Not sure…. 3
1.9	Do you feel that you are over-worked?	Yes …..1<br>No ….. 2

2. TRAINING

2.1 CURRENT TRAINING

	Question	Response
2.1.1	Do you do or are you involved in any of the following services?<br>*[Read the service and circle all given answers]*	STI…..1<br>Family Planning…..2<br>VCT…..3<br>ANC/midwifery/post-natal …..4<br>PMTCT…..5<br>PHC….. 6<br>HIV….. 7<br>TOP….. 8<br>Other (specify):__________________…. 77
2.1.2	Have you been trained in any of the following in the last 2 years? *[Read the service and circle all given answers]*	Family Planning…..1<br>ARV…..2<br>VCT/HIV counseling…..3<br>HIV management…..4<br>PMTCT…..5<br>STI…..6<br>TB…..7<br>ANC/midwifery/post-natal…..8<br>Sexual health…..9<br>Taking sexual history….10<br>TOP….11<br>Values clarification….12<br>Other (specify):__________________…. 77

2.1.3	Please list areas in which you would like more experience/training?<br>*[Circle all given answers]*	Family Planning…..1<br>ARV…..2<br>VCT/HIV counseling…..3<br>HIV management…..4<br>PMTCT…..5<br>STI…..6<br>TB…..7<br>ANC/midwifery/post-natal…..8<br>Sexual health…..9<br>Taking sexual history….10<br>TOP….11<br>Values clarification….12<br>Other (specify)___________________....77

2. 2 SEXUALITY TRAINING, CLIENT CONDOM USE AND RISK REDUCTION COUNSELING

	Question	Response	Skip
2.2.1	Do you record sexual history information from your clients? (e.g. whether client is sexually active, number of partners, etc)	Yes ….1<br>No…..2<br>Depends on client/consultation…3	
2.2.2	How do you/would you feel about taking a client's sexual history?	Very comfortable ….. 1<br>Comfortable….. 2<br>Uncomfortable….. 3<br>Very uncomfortable …..4	
2.2.3	Are there any types of clients that you might feel uncomfortable taking a sexual history from or discussing their sexual behaviour? *[Choose all that provider mentions]*	No group…..1<br>Married women….. 2<br>Married men….. 3<br>Adolescent girls ….. 4<br>Adolescent boys….. 5<br>Other (specify)________________<br>______________________________<br>_______________________…..77	
2.2.4	How do you feel about counseling and examining HIV positive clients?	Very comfortable ….. 1<br>Comfortable….. 2<br>Uncomfortable…..3<br>Very uncomfortable….. 4<br>Do not counsel clients…..5<br>Do not examine HIV positive clients….6	

2.2.5	In the last month, how often did you discuss condoms/condom use with your clients in a one to one consultation?	Never….. 1<br>Less than half the clients….. 2<br>Half the time….. 3<br>More than half…..4<br>Almost all/all…..5	
2.2.6	Have you been trained on the female condom?	Yes …..1<br>No …..2	
2.2.7	Have you ever shown a client how to use a female condom?	Yes…..1<br>No…..2	
2.2.8	Have you shown any client how to use a male condom in last 3 months?	Yes…..1<br>No…..2	
2.2.9	How do you make male condoms available to the client?	I give them to client…..1<br>Dispenser in facility…..2<br>Other (specify):___________….77	

2.2.10	When you talk about male condoms, what do you normally discuss with the client?<br>*[Circle all that apply]*	Tell them how to use the condom…..1<br>Show them how to use the condom….2<br>Mention of dual protection (protection against pregnancy and STIs/HIV)…..3<br>VCT…..4<br>STI and HIV risk….. 5<br>Importance of correct and consistent use….6<br>Negotiation of condom use….. 7<br>Gender-based violence …. 8<br>Lubricant…..9<br>ECPs…..10<br>What to do if it breaks.....11<br>Storage....12<br>Expiry date.....13<br>Other (specify):________________<br>______________________________<br>_________________________….77	
2.2.11	Have you personally given male condoms to any client in the last 3 months? (not just told them they are in dispenser)	Yes…..1<br>No…..2	
2.2.12	Have you heard anything about the potential for male circumcision to prevent HIV and other STIs?	Yes…..1<br>No…..2	If no, go to 2.2.14

2.2.13	What have you heard?<br>*[Circle all that apply]*	Reduces the risk of STI infection…..1<br>Reduces the risk of AIDS/HIV infection…..2<br>Improves Hygiene …..3<br>Improves sex for the man…..4<br>Improves sex for the woman…..5<br>Other (specify)________________<br>______________________________<br>_________________________….77	
2.2.14	Do you think health facilities should offer male circumcision as an option for men?	Yes…..1<br>No…...2<br>Not sure….. 3	If no/not sure, go to next section (3)

2.2.15	If yes, which health care facilities should provide the service?<br>*[Circle all that apply]*	Clinics…..1<br>Community health centres…..2<br>Hospitals…..3<br>Other (specify)____________....77	

3. CURRENT PRACTICES

3.1 FAMILY PLANNING

Providers who work in TOP should also respond to this section.

	Question	Response	Skip
3.1.1	Do you see clients who have come to your health facility for family planning?<br>*[If on rotation and has not seen for 6 months we can still include them here]*	Yes…..1<br>No…..2	If no, go to next section (3.2)

3.1.2	What kinds of methods of contraception are discussed with new clients?<br>*[Circle all the provider mentions]*	Injectable contraceptives …..1<br>Combined oral contraceptives (COCs)….. 2<br>Progestin-only oral contraceptives (POPs)….. 3<br>Male condoms….. 4<br>Female condoms….. 5<br>Intrauterine devices (IUDs)…..6<br>Emergency contraception…..7<br>Tubal ligation…..8<br>Vasectomy….. 9<br>Other (specify):______________________<br>_______________________________….77	
3.1.3	Do you ever see clients who are less than 16 years old?	Yes…1<br>No…2	If no, go to 3.1.6
3.1.4	Did you give contraception to the last client under 16 years old that you saw for family planning?	Yes …..1<br>No ….. 2<br>Can't remember….. 3	If no/can't remember, go to 3.1.6

3.1.5	**If yes**, what did you give?<br>*[Circle all that provider mentions]:*	Injectable 2-month…..1<br>Injectable 3-month….. 2<br>Combined oral contraceptives (COCs)….. 3<br>Progestin-only oral contraceptives (POPs)….. 4<br>Male condoms…..5<br>Female condom….. 6<br>Other (specify):______________________<br>_______________________________….77	
3.1.6	Have you referred a woman for a TOP (termination of pregnancy) in the last year?	Yes…..1<br>No …..2	

3.1.7	If a client is over two weeks late for her injection or late for pill resupply (has missed pills for plus one day), what do you do?<br>*[Circle all that apply]*	Check she is not pregnant and give her pills/injection…..1<br>Ask her to return when she is menstruating…..2<br>Prescribe emergency contraception if had unprotected sex in last 72 hours/3 days….3<br>Other (specify)______________________<br>____________________________________<br>_______________________________….77	

3.1.8	Approximately how many IUDs have you fitted in the last 6 months?	Number______________<br>Not applicable (IUD not available in facility)….88	If has fitted some, go to 3.1.11
3.1.9	**If IUD not available** in facility how many clients have been referred?	Number___________________________	
3.1.10	**If IUD not available** where were/would clients be referred?		
3.1.11	Are there certain clients who you would consider **not** suitable for an IUD?	Yes…..1<br>No….. 2	If no, go to 3.1.13
3.1.12	**If yes**, which of the following would not be suitable for an IUD?<br>*[Read out and circle all mentioned]*	Under 25…..1<br>Nulliparous (never pregnant)….. 2<br>At risk of STIs…..3<br>At risk of HIV/HIV positive…..4<br>Not in steady relationship…..5<br>Other (specify)______________________<br>____________________________________<br>________________________________….77	

3.1.13	Have you ever discussed HIV testing with a family planning client?	Yes…1<br>No…2	If no, go to 3.1.21
3.1.14	Do you routinely offer HIV tests to *all* family planning clients?	Yes…..1<br>No…..2	If yes, go to 3.1.16
3.1.15	**If no,** what would prompt you to offer an HIV test to a woman or a man coming in for family planning?<br>*[Circle all the provider mentions:]*	If client asks for it…..1<br>I offer it to everyone…..2<br>Patient presents with STI or OI…..3<br>Patient's partner/child is known positive…..4<br>Patient is having unprotected sex…..5<br>Other (specify)__________________….77	
3.1.16	Have you personally done an HIV test to a family planning client in last 3 months?	Yes…..1<br>No…..2	If no go to 3.1.18

3.1.17	**If yes,** have you offered to both men and women?	Men only…..1<br>Women only…..2<br>Both, men and women…..3	
3.1.18	How many family planning clients have you offered HIV tests to in the last 3 months?	Number (approx)_____________________	

3.1.19	Do you actually do HIV testing yourself, or do you refer for testing?	Yes, I do HIV testing…..1<br>I refer to VCT counselors in this facility…2<br>I refer to VCT site not in this facility…3<br>Depends on circumstances…..4<br>I	
3.1.20	Are clients advised to return for a repeat test after the window period?	Yes…1<br>No….2	
3.1.21	Have you personally given emergency contraception to a client in the last 3 months?	Yes …..1<br>No …...2	
3.1.22	Have you ever performed a pap smear in this current post?	Yes …..1<br>No …...2	If yes, go to 3.1.24
3.1.23	**If not**, why not?<br>*[Circle all that apply]*	Not trained….1<br>No equipment here to do pap smears….2<br>Clients rarely get their results.…3<br>Results get lost in the system.…4<br>There are not enough specula here.…5<br>I am new to this position.…6<br>We refer clients elsewhere for pap smears (specify where ____________________ ______________________________) …7	
3.1.24	Under what conditions would you offer or perform a pap smear?<br>*[Circle all the provider mentions]*	Woman over 30…..1<br>HIV+…..2<br>Client with STI…..3<br>Patient requests one…..4<br>Sexually active…..5<br>Family history of cancer…..6<br>Never perform.…7<br>Other (specify)______________________ ________________________________….77	
3.1.25	If you know a family planning client is HIV positive, do you discuss any of these issues in the consultation?<br>*[Read out and circle all]*	Should be using condoms/dual protection…..1<br>Don't mention HIV status unless client does….. 2<br>Ask if they are in wellness/ART programme…..3<br>Other(specify)_______________________ ______________________________ ….77	

3.1.26	What contraceptive method would you recommend to an HIV positive client?	Injectables only…..1<br>Injectables and condoms…..2<br>COC Pills only…..3<br>COC Pills and condoms…..4<br>POP pills only…..5<br>POP pills and condoms…..6<br>Condoms only…..7<br>Sterilization and condoms…..8<br>Sterilization only…..9<br>IUD….10<br>IUD and condoms....11<br>Other (specify)______________________<br>______________________________….77	
3.1.27	Do you think there are any drug interactions between ARVs and contraception?	Yes…..1<br>No…..2<br>Don’t know /not sure….88	If no or DK go to 3.1.29
3.1.28	**If yes**, please explain.	Interferes with hormonal injection…..1<br>Interferes with COCs (decreases efficacy)….. 2<br>Need to come back earlier for injection…..3<br>Other (specify)______________________<br>__________________________________<br>______________________________….77	

3.1.29	Do you think a healthy HIV positive woman should have children?	Yes…1<br>Yes, if she has no children…..2<br>Yes, even if she has children…..3<br>No….4<br>No, even if she has no children…..5<br>No, she should not have them even if she has children already…..6	
3.1.30	If a client complains of itching or discharge in her family planning consultation what do you do?<br>*[Do not read out, circle all mentioned]*<br>*If does not mention examination, ask “do you examine”?*<br>*Probe- anything else?*	Always do a physical exam and then treat…..1<br>Sometimes do a physical exam and treat…..2<br>Don’t examine, treat on verbal symptoms…..3<br>Recommend an HIV test if status unknown…..4<br>Tell her to go to PHC/another service…..5<br>Counsel on condoms …..6<br>Other (specify)_________________….77	

3.1.31	If a family planning client asks for, or needs STI treatment and asks for an HIV test, which of the 3 services will you usually see her for?	FP, STI & VCT …..1<br>FP & STI, refer for VCT to counselors …..2<br>FP & VCT, refer for STI …..3<br>FP only …..4	If all 3 services, go to next section (3.2)
3.1.32	**If not all 3 services**, why can she not have all three services with you?<br>*[Circle all that apply]*	I am too busy to do all 3…..1<br>Counselors always do VCT…..2<br>ST treatment done in PHC/elsewhere…..3<br>I have not recently been trained/ nor am I adequately trained in VCT/STI…..4<br>Other (Specify)_____________________<br>________________________________<br>______________________________….77	

3.2 VOLUNTARY COUNSELING AND TESTING (VCT)

All providers to respond.

	Question	Response	Skip
3.2.1	What is your role in VCT in your facility?<br>*[Circle all that apply]*	Counsel…..1<br>Conduct rapid HIV test..…2<br>Confirmatory test (nurses)…..3<br>Take blood for CD4…..4<br>Refer to VCT counselor/other staff…..5<br>Not involved…..6<br>Management role…..7<br>Other (specify)________________….77	
3.2.2	Which clients in this facility get offered HIV testing routinely? (routinely means always get offered if they don't know their status)<br>*[Read out]*	ANC clients…..1<br>STI clients…..2<br>FP clients…..3<br>TB patients…..4<br>PHC clients…..5<br>Postnatal clients…..6<br>TOP clients…..7<br>Don't know…..88<br>Other (specify)________________….77	
3.2.3	From what age should people routinely start being offered HIV tests?	No minimum age….1<br>Minimum age:___________________	
3.2.4	Do you believe that there is an age that is too old to get an HIV test?	Yes.....1<br>No..…2	If no, go to 3.2.6
3.2.5	**If yes**, what age is it?	Maximum age:___________________	

3.2.6	Does an informed consent get taken from each person tested?	Yes…..1<br>No …..2<br>Don't know….88	
3.2.7	Do patients have the opportunity to decline testing?	Yes…..1<br>No …..2<br>Don't know….88	

3.2.8	If a patient declines to get tested, what are reasons a patient may give for why?<br>*[Circle all that apply]*	Scared of getting positive results…..1<br>Already tested negative before…..2<br>Partner does not want client to take test…..3<br>Does not want to know status…..4<br>Thinks there is nothing that can be done about HIV…..5<br>She/he knows/suspects being HIV+…..6<br>Is worried about confidentiality…..7<br>Thinks not at risk from HIV…8<br>Other (specify)____________….77<br>Do not know….88			
3.2.9	Do you counsel/do VCT yourself?	Yes…..1<br>No …..2			If no, go to next section (3.3)
**If yes,** when you counsel a patient on HIV testing, what is discussed in:		**Pretest counseling**	**Post test counsel-ing: Positive**	**Post test counsel-ing: Negative**	**Ongoing counsel-ing**
3.2.10	Risk reduction, including safer sex/condoms	Yes…..1<br>No…..2	Yes…..1<br>No…..2	Yes…..1<br>No…..2	Yes…..1<br>No…..2
3.2.11	Faithfulness/partner reduction	Yes…..1<br>No…..2	Yes…..1<br>No…..2	Yes…..1<br>No…..2	Yes…..1<br>No…..2
3.2.12	PMTCT	Yes…..1<br>No…..2	Yes…..1<br>No…..2	Yes…..1<br>No…..2	Yes…..1<br>No…..2
3.2.13	How to disclose to others, including partner	Yes…..1<br>No…..2	Yes…..1<br>No…..2	Yes…..1<br>No…..2	Yes…..1<br>No…..2
3.2.15	ARV and other treatment options	Yes…..1<br>No…..2	Yes…..1<br>No…..2	Yes…..1<br>No…..2	Yes…..1<br>No…..2
3.2.16	Window period	Yes…..1<br>No…..2	Yes…..1<br>No…..2	Yes…..1<br>No…..2	Yes…..1<br>No…..2
3.2.17	Positive living	Yes…..1<br>No…..2	Yes…..1<br>No…..2	Yes…..1<br>No…..2	Yes…..1<br>No…..2
3.2.18	TB	Yes…..1<br>No…..2	Yes…..1<br>No…..2	Yes…..1<br>No…..2	Yes…..1<br>No…..2

**If yes,** when you counsel a patient on HIV testing, what is discussed in:		**Pretest counseling**	**Post test counsel-ing: Positive**	**Post test counsel-ing: Negative**	**Ongoing counsel-ing**
3.2.19	STIs	Yes…..1<br>No…..2	Yes…..1<br>No…..2	Yes…..1<br>No…..2	Yes…..1<br>No…..2
3.2.20	Contraception/family planning	Yes…..1<br>No…..2	Yes…..1<br>No…..2	Yes…..1<br>No…..2	Yes…..1<br>No…..2
3.2.21	Domestic violence	Yes…..1<br>No…..2	Yes…..1<br>No…..2	Yes…..1<br>No…..2	Yes…..1<br>No…..2
3.2.22	Stigma/discrimination	Yes…..1<br>No…..2	Yes…..1<br>No…..2	Yes…..1<br>No…..2	Yes…..1<br>No…..2
3.2.23	Grant assistance from government	Yes…..1<br>No…..2	Yes…..1<br>No…..2	Yes…..1<br>No…..2	Yes…..1<br>No…..2
3.2.24	CD4 count/viral load	Yes…..1<br>No…..2	Yes…..1<br>No…..2	Yes…..1<br>No…..2	Yes…..1<br>No…..2
3.2.25	Reinfection	Yes…..1<br>No…..2	Yes…..1<br>No…..2	Yes…..1<br>No…..2	Yes…..1<br>No…..2
3.2.26	TOP	Yes…..1<br>No…..2	Yes…..1<br>No…..2	Yes…..1<br>No…..2	Yes…..1<br>No…..2
3.2.27	Nutrition	Yes…..1<br>No…..2	Yes…..1<br>No…..2	Yes…..1<br>No…..2	Yes…..1<br>No…..2
3.2.28	Other (specify)__________________	Yes…..1<br>No…..2	Yes…..1<br>No…..2	Yes…..1<br>No…..2	Yes…..1<br>No…..2

	**Question**	**Response**
3.2.29	Wellness: If a woman or a man tests positive but the CD4 count is >200, what wellness management issues do you discuss with client?<br>*[Circle all that apply]*	Safer sex…..1<br>Treating opportunistic infections (with emphasis on TB)…..2<br>Signs of deteriorating health…..3<br>When to return for CD4 blood count…..4<br>Getting sexual partners/children tested…..5<br>Nutrition/hydration…..6<br>Treatment options…..7<br>PMTCT…..8<br>Family planning…..9<br>Emergency contraception….10<br>Fertility intentions….11<br>TOP….12<br>Possible violence on disclosure….13<br>Exercise….14<br>Reinfection….15<br>Other (specify)____________________________….77

3.2.30	If a woman or a man tests positive and the CD4 count is <200, what do you discuss with client?<br>*[Circle all that apply]*	Referral to other facilities…..1<br>Treatment options…..2<br>Enrolling in treatment literacy…..3<br>Safer sex…..4<br>Treating opportunistic infections (with emphasis on TB)…..5<br>Signs of deteriorating health…..6<br>When to return for CD4 blood count…..7<br>Getting sexual partners/children tested…..8<br>Nutrition/hydration…..9<br>PMTCT….10<br>Family planning….11<br>Emergency contraception….12<br>Fertility intentions….13<br>TOP….14<br>Possible violence on disclosure….15<br>Exercise….16<br>Reinfection….17<br>Don't know….88<br>Other (specify)__________________________….77

3.3 HIV SERVICES (TREATMENT/ARVs, WELLNESS)

All providers to respond

	Question	Response	Skip
3.3.1	Do you see clients who come for HIV services?	Yes…..1<br>No….. 2	
3.3.2	Do you feel comfortable identifying and treating AIDS-related illnesses/opportunistic infections?	Yes…..1<br>No….. 2<br>Don't know…. 88	
3.3.3	Is there a system for fast tracking priority clients for ARV in your facility?	Yes…..1<br>No….. 2<br>Don't know…. 88	If no or DK go to 3.3.5
3.3.4	**If yes**, please describe.		

3.3.5	What kind of clients would you consider eligible for fast track/priority for ARV?<br>*[Circle all that provider mentions]:*	Infants and children…..1<br>Pregnant women…..2<br>Low CD4 count <50…..3<br>AIDS defining condition- Stage 4…..4<br>Very sick patients…5<br>Patients with TB…6<br>Don't know….88<br>Other (specify)____________________….77	
3.3.6	Do you think that healthy HIV+ women should have children?<br>*[do not ask if responded in Q 3.1.28]*	Yes…..1<br>Yes, if she has no children…..2<br>Yes, even if she has children…..3<br>No…..4<br>No, even if she has no children…..5<br>No, she should not have them even if she has children already…..6<br>Don't know….88<br>Other (specify)_____________________...77	
3.3.7	Do you think there are any interactions between ARV and contraception? *[Do not ask if already responded in Q 3.1.27]*	Yes…..1<br>No…..2<br>Don't know /not sure….88	If no or DK go to 3.3.9
3.3.8	**If yes**, please explain.	Interferes with hormonal injection…..1<br>Interferes with COCs (decreases efficacy)….. 2<br>Need to come back earlier for injection…..3<br>Other (specify)____________________….77	
3.3.9	Are clients who are not in possession of a South African ID document allowed access to HIV services, including ARV?	Yes…..1<br>No….. 2<br>Don't know ….88	
3.3.10	Do you provide family planning services to HIV positive clients?	Yes, counseling and give method…..1<br>Counseling only…….2<br>No counseling or method given…..3<br>Don't know….88<br>Other (Specify)_________________...77	

3.3.11	If an HIV positive client is not using family planning, what do you advise?	Should be sterilised….. 1<br>Should use hormonal injection….. 2<br>Should use COCs…..3<br>Should use condoms and hormonal contraception…..4<br>Should use IUD…..5<br>Should use IUD and condoms…..6<br>Don't know….88<br>Other (specify)___________________….77	
3.3.12	Have you ever performed a pap smear in this current post?	Yes …..1<br>No …...2	If yes, go to next section (3.4)
3.3.13	If not, why not?<br>[Circle all that apply]	Not trained….1<br>No equipment here to do pap smears….2<br>Clients rarely get their results….3<br>Results get lost in the system….4<br>There are not enough specula here….5<br>I am new to this position….6<br>We refer clients elsewhere for pap smears (specify where ____________________<br>_____________________________) …7<br>Other (specify)_______________________<br>_______________________________....77	

3.4 PRIMARY HEALTH CARE (PHC)

	Question	Response	Skip
3.4.1	Do you see clients who come for PHC services?<br>*[Include those providers who have seen them in the last year]*	Yes…..1<br>No ….. 2	If no, go to next section (3.5)
3.4.2	Have you ever discussed HIV testing with a primary health care client?	Yes…1<br>No…2	If no, go to 3.4.9
3.4.3	Do you routinely offer HIV tests to *all* primary health care clients?	Yes…..1<br>No…..2	

3.4.4	What would prompt you to offer an HIV test to a woman or a man coming in for PHC?<br>*[Circle all that apply]*	Client asked for a test …..1<br>Presented with symptoms of STI/HIV…..2<br>Client has TB…..3<br>Client has persistent cough…..4<br>Diarrhoea …..5<br>Weight loss…..6<br>Client is sick..…7<br>Other (specify)___________________….77	
3.4.5	Have you personally done an HIV test to a primary health care client in last 3 months?	Yes…..1<br>No…..2	If no go to 3.4.7
3.4.6	If yes, have you offered to both men and women?	Men only…..1<br>Women only…..2<br>Both, men and women…..3	
3.4.7	How many primary health care clients have you offered HIV tests to in the last 3 months?	Number (approx)_____________________	
3.4.8	Do you actually do HIV testing yourself, or do you refer for testing?	Yes, I do HIV testing…..1<br>I refer to VCT counselors in this facility…2<br>I refer to VCT site not in this facility…3<br>Depends on circumstances…..4	
3.4.9	Are clients advised to return for a repeat test after the window period?	Yes…1<br>No….2	
3.4.10	How would you deal with a client complaining of a persistent cough over several weeks?<br>*[Do not prompt]*	TB test…..1<br>TB test not mentioned…..2<br>Other (specify)____________________….77	If TB test not mention-ed, go to 3.4.14
3.4.11	Would you do the TB test or refer to another service point for TB test?	Yes, I would do the test…..1<br>No, refer for test<br>(specify where)____________________…..2	If no, go to 3.4.14
3.4.12	If positive for TB, is treatment done in PHC or another clinic/service?	Yes, treatment in PHC…1<br>No, refer to other service in clinic…2	If no, go to 3.4.14
3.4.13	**If yes**, please specify, where client would be sent:		

3.4.14	Please specify what indications allow for a patient to be tested or treated for TB?	
3.4.15	If a client is offered a TB test are they also offered an HIV test?	Yes, if they are positive or negative for TB…..1<br>Yes, if they are positive for TB…..2<br>No, not offered HIV test unless they ask…..3<br>Other (specify)____________________....77<br>Don't know….88

3.5 STI SERVICES

	Question	Response	Skip
3.5.1	Do you see clients who come for STI services (can be in PHC or in FP)?<br>*[If provider has seen them in last year, include]*	Yes…..1<br>No….. 2	If no, go to next section (3.6)
3.5.2	When you counsel a patient who has an STI, what do you normally discuss?<br>*[Circle all that provider mentions]*	HIV/AIDS…..1<br>VCT…..2<br>Risk of having multiple partners…..3<br>Abstinence during STI treatment…..4<br>Partner referral for STI treatment…..5<br>Family planning…..6<br>Condom use…..7<br>Other (specify)___________________<br>___________________________….77	
3.5.3	Have you ever discussed HIV testing with an STI client?	Yes…1<br>No…2	If no, go to 3.5.11
3.5.4	Do you routinely offer HIV tests to *all* STI clients?	Yes…..1<br>No…..2	If yes, go to 3.5.6

3.5.5	**If no,** what would prompt you to offer an HIV test to a woman or a man coming in with an STI?<br>*[Circle all that provider mentions]*	Client asks for it…..1<br>I offer it to clients who have repeat STIs….2<br>Patient's partner/child is known positive…..3<br>Other (specify)__________________<br>__________________________….77	
3.5.6	Have you offered a client with STIs a HIV test in the last 3 months?	Yes …..1<br>No…..2	If no go to 3.5.8
3.5.7	**If yes**, have you offered to both men and women?	Men only…..1<br>Women only…..2<br>Both, men and women…..3	
3.5.8	How many STI clients have you offered HIV tests to in the last 3 months?	Number (approx)________________	

3.5.9	Do you actually do HIV testing yourself, or do you refer for testing?	Yes, I do HIV testing…..1<br>I refer to VCT counselors in this facility…2<br>I refer to VCT site not in this facility…3<br>Depends on circumstances…..4	
3.5.10	Are clients advised to return for a repeat test after the window period?	Yes…1<br>No…2	
3.5.11	Are male condoms kept in the consultation rooms to give directly to clients?	Yes, all consulting rooms…..1<br>Yes, some consulting rooms…..2<br>No, in dispensers only…..3	
3.5.12	Do you give STI clients condoms in the consultation room?	Yes, always in consultation…..1<br>Tell them to get from facility dispenser…..2<br>Don't discuss where to get them…..3<br>Other (specify)__________________<br>__________________________….77	
3.5.13	Do you do have a system for contact tracing for STIs in place?	Yes…..1<br>No….. 2	
3.5.14	Do you usually discuss contraception with STI clients?<br>*[Circle all that provider mentions]*	Yes, always…..1<br>Yes, sometimes…..2<br>Depends on the client…..3<br>If the client asks…..4<br>Usually not…..5<br>Other (specify)__________________<br>______________________________<br>__________________________….77	

3.5.15	Do you counsel on the importance of finishing a full course of treatment?	Yes…..1<br>No….. 2	

3.6 ANC (ANTENATAL CLINIC) / PMTCT

	Question	Response	Skip
3.6.1	Do you see clients who come for ANC services?	Yes…..1<br>No….. 2	If no, go to next section (3.7)
3.6.2	What ANC topics are normally covered by group talks?	Health talks…..1<br>PMTCT talk (HIV test)…..2<br>Other (specify)________________________<br>________________________________….77	
3.6.3	Have you ever discussed HIV testing with an ANC client?	Yes…1<br>No…2	If no, go to 3.6.11
3.6.4	Do you routinely offer HIV tests to *all* ANC clients?	Yes…..1<br>No…..2	If yes, go to 3.6.6
3.6.5	**If no,** what would prompt you to offer an HIV test to a pregnant woman?<br>*[Circle all that apply]*	Client asks for it…..1<br>I offer it to clients who have had STIs….2<br>Patient's partner/child is known positive…..3<br>Other (specify)________________________<br>________________________________….77	
3.6.6	When during a woman's pregnancy do you offer an HIV test?<br>*[Circle all that apply]*	First presentation at the ANC clinic…..1<br>Subsequent ANC visits…..2<br>At the time of delivery (or within 72 hours after birth)…..3<br>At postnatal care…..4<br>Well-baby clinic visits…..5<br>Other (specify)____________________….77	
3.6.7	Do you offer pregnant women, who tested HIV negative, another HIV test later/towards the end of pregnancy?	Yes…..1<br>No…..2<br>Sometimes…..3	
3.6.8	Do you offer VCT more than once so that a pregnant woman who has previously declined VCT has a chance to reconsider?	Yes…..1<br>No…..2	If no, go to 3.6.10
3.6.9	**If yes,** when do you usually do repeat HIV testing during pregnancy?	Write gestational weeks:______________	

3.6.10	Do you actually do HIV testing yourself, or do you refer for testing?	Yes, I do HIV testing…..1<br>I refer to VCT counselors in this facility…2<br>I refer to VCT site not in this facility…3<br>Depends on circumstances…..4	
3.6.11	What are the reasons some pregnant women refuse to test for HIV?<br>*[Circle all that apply]*	They are scared of getting positive results…..1<br>They do not want to know their status…..2<br>They feel there is nothing that can be done about HIV…..3<br>Some know/ suspect that they are HIV+…..4<br>Other (specify)___________________….77	
3.6.12	What is your facility doing on the prevention of unwanted unplanned pregnancy for HIV positive clients<br>*[Probe for more and circle all given answers]*	Counsel them on FP….1<br>Counsel them on dual protection….. 2<br>Counsel them on CTOP….. 3<br>Other (specify)________________________<br>_____________________________________<br>________________________________….77	

3.6.13	Who normally does this counseling?	Lay counselors….. 1<br>Both nurses and lay counselors…..2<br>Nurses…..3<br>Other (specify)___________________….77	
3.6.14	If a pregnant woman less than 12 weeks gestation finds out she is HIV positive and asks for a TOP, would you refer her to a TOP service/facility/clinic?	Yes…..1<br>No…..2<br>Depends on the client..…3<br>Other (specify)____________________…..4	
3.6.15	Many HIV positive mothers are lost and not followed up after delivery: why do you think this may be so?<br>*[Circle all that apply]*	They feel stigmatised/They do not want to anyone to know they are HIV+…..1<br>They did not know they needed to come back …..2<br>They don't actually live in the area…..3<br>Other (specify)___________________….77	
3.6.16	Is family planning routinely discussed with pregnant women or new mothers?	Yes…1<br>No…2	If no, go to next section (3.7)
3.6.17	When is family planning discussed with pregnant women or new mothers?	Before delivery…..1<br>At hospital…..2<br>At post natal visit…..3<br>It is not generally discussed/never…..4<br>Other (specify)___________________….77	

3.6.18	What is the mother told about infant feeding practices if she is HIV positive?	Exclusive breast feeding for 6 months…..1<br>Exclusive formula feeding for 6 months…..2<br>It depends on circumstances…3<br>Both 1 & 2, client advised to choose best option…..4<br>Nothing, this is covered in Labour/Postnatal/Wellbaby clinic…..5<br>Other (specify)_________________….77	
3.6.19	In what circumstances would a woman be counseled to do exclusive breastfeeding? *[Circle all that apply}*	Infant formula not acceptable culturally…1<br>Stigma from formula use…2<br>Mother does not have time/skills/knowledge to use formula…3<br>Formula not affordable…4<br>No access to clean water…5<br>No regular access to formula…6<br>Mother unable to prepare formula safely/hygienically…7<br>Other (specify)______________________<br>_____________________________...77	
3.6.20	How do you manage women who test HIV positive during pregnancy? *[Circle all that apply]*	Do CD4 count…..1<br>Do WHO Clinical staging…..2<br>Encourage partner to test…..3<br>Enroll them on the PMTCT programme..…4<br>Refer to the PMTCT programme at Wentworth hospital….3<br>Refer to PMTCT elsewhere (specify)_____________________.....4<br>Provide ARVs during pregnancy at this facility..…5<br>No action taken..….6<br>Other (specify)________________________<br>___________________________________<br>_________________________________....77	If answers 5, go to next question, otherwise skip to next section
3.6.21	What regimen do you put women with a high CD4 count (over 200) on during pregnancy? *[Circle all that apply]*	AZT (from 28 weeks)…..1<br>AZT other time (specify)____________.....2<br>NVP…..3<br>Don't know….88<br>Other (specify)__________________….77	

3.6.22	What regimen do you put women with a low CD4 count (less than 200) on during pregnancy?<br>*[Circle all that apply]*	d4T + 3TC + NVP(1b)…..1<br>d4T + 3TC + EFV (1a)…..2<br>Depends on pregnancy stage (specify)__<br>___________________________________<br>________________________________...3<br>Don't know…...88<br>Other (specify)___________________….77	
3.6.23	Under what circumstances is co-trimoxazole prophylaxis given to pregnant women?	CD4 <200..…1<br>WHO clinical stage IV..…2<br>Both 1 & 2 ..…3<br>To all HIV positive pregnant women..…4<br>Other (specify)_____________....77	

3.7 DELIVERY CARE/IMMEDIATE POST-NATAL CARE/PMTCT (IN HOSPITAL)

This section is about labour/delivery and post-natal care. The post-natal care is a post delivery check up for the mother in the hospital. We want to find out what the procedures and examinations involved are.

	Question	Response	Skip
3.7.1	Do you work in the delivery ward and in post-natal care?	Yes…..1<br>No…..2	If no, go to next section (3.8)
3.7.2	How do you identify HIV positive pregnant women when they come for delivery?	**Describe system:**	
3.7.3	Does the mother get offered an HIV test during labour if she did not have one during pregnancy?	Yes…..1<br>No…..2	
3.7.4	What drugs are given to HIV positive women with a high CD4 count (over 200) during labour and delivery?<br>*[Circle all that apply]*	AZT.....1<br>NVP…..2<br>Don't know….88<br>Other (specify)_________________….77	

3.7.5	What drugs do you put HIV positive women with a low CD4 count (less than 200) on during labour and delivery?<br>*[Circle all that apply]*	d4T + 3TC + NVP(1b)…..1<br>d4T + 3TC + EFV (1a)…..2<br>NVP…..3<br>AZT…..4<br>Don't know…...88<br>Other (specify)__________________….77	

3.7.6	When are the women in labour/delivery normally given this/these drug(s)?	Before labour begins….1<br>Onset of labour…2<br>Onset of labour and 3 hourly…..3<br>When baby is born…4<br>Depends on regimen (specify)____<br>___________________________.....5<br>Another time (specify)________________<br>______________________________...77	
3.7.7	Which antiretroviral drugs do newborns usually take?	Nevirapine syrup…1<br>AZT syrup….2<br>Both…3<br>It depends (specify)________________<br>______________________________….4<br>Other(specify)________________...77	If mention NVP, go to 3.7.8<br>If mention AZT, go to 3.7.9, others to 3.7.10
3.7.8	**If mention nevirapine:** When do newborns normally take the nevirapine syrup?	Immediately after birth…1<br>Within 6 hours of birth…2<br>After 1 day…3<br>After 2 days…4<br>Within 3 days…5<br>Other (specify)__________________...77	
3.7.9	**If mention AZT:** under what circumstances do newborns take AZT syrup?<br>*[Circle all that apply]*	All infants receive AZT…1<br>Take for 4 weeks if mother receives < 4 weeks of AZT or HAART during pregnancy….2<br>Take for 1 week if mother receives > 4 weeks of AZT or HAART during pregnancy….3<br>Other (specify) __________________...77<br>Don't know….88	
3.7.10	How long after a normal delivery is a woman monitored for?	24 hours….1<br>Other(specify)_____________________77	

3.7.11	How long after a C-section is a woman monitored for?	2 days…1<br>3 days….2<br>Other (specify)___________________77	
3.7.12	Does the baby get a physical exam during post-natal care at the hospital?	Yes…..1<br>No….. 2	

3.7.13	Does the mother get a physical exam during post-natal care at the hospital?	Yes…..1<br>No…..2	
3.7.14	What is the mother told about infant feeding practices if she is HIV positive?	Exclusive breast feeding for 6 months…..1<br>Exclusive formula feeding for 6 months…..2<br>It depends on circumstances…3<br>Both 1 & 2, client advised to choose best option…..4<br>Nothing, this is covered in ANC/Postnatal/Wellbaby clinic…..5<br>Other (specify)_________________….77	

3.7.15	In what circumstances would a woman be counseled to do exclusive breastfeeding? *[Circle all that apply}*	Infant formula not acceptable culturally…1<br>Stigma from formula use…2<br>Mother does not have time/skills/knowledge to use formula…3<br>Formula not affordable…4<br>No access to clean water…5<br>No regular access to formula…6<br>Mother unable to prepare formula safely/hygienically…7<br>Other (specify)_____________________<br>_________________________________<br>______________________________...77	
3.7.16	What other counselling is given to HIV positive women who have recently given birth?<br>*[Circle all that apply]*	Infant feeding techniques….1<br>Skin-to-skin contact…2<br>Advised for infant HIV test at 6 weeks…3<br>Need for baby to start co-trimoxazole prophylaxis at 6 weeks…4<br>Come back for postpartuml/well baby visit at 6 weeks…..5<br>Other (specify)_____________...77	

3.7.17	Is there a system for tracking women in the PMTCT program during delivery and after delivery?	Yes …..1<br>No….. .2	If no, go to next section (3.8)
3.7.18	**If yes**, please describe:		
3.7.19	Do you routinely check for a woman's CD4 count at delivery/post natally?	Yes…1<br>No….2	

3.8 WELL BABY/POST PARTUM CARE/PMTCT

This section is about post partum care. We want to find out if women and their babies come for these visits and if they do, what are the procedures and examinations involved.

	Question	Response	Skip
3.8.1	Do you see clients who come for well baby/post-partum care services?	Yes…..1<br>No…..2	If no, go to next section (4)
3.8.2	When should a woman come for a well baby /post-partum visit?	In first 7 days post delivery…..1<br>Within two weeks of delivery….. 2<br>Six weeks after delivery….3<br>Depends (specify on what)_____________<br>_________________________________...66<br>Other time (specify)_______________….77	
3.8.3	When should an HIV Positive woman come for a well baby/post-partum visit?	In first 7 days post delivery…..1<br>Within two weeks of delivery….. 2<br>Six weeks after delivery….3<br>Every week during the first month…4<br>Depends (specify on what)_____________<br>_________________________________...66<br>Other time (specify)_______________….77	
3.8.4	Do women generally come for a post-partum visit or is it usually combined with baby immunization visit?	Yes, come for a post partum visit independently of immunization…..1<br>Usually combined with an immunization visit….. 2<br>Other (specify)____________________….77	

3.8.5	What is the mother told about infant feeding practices if she is HIV positive?	Exclusive breast feeding for 6 months…..1<br>Exclusive formula feeding for 6 months…..2<br>It depends on circumstances…3<br>Both 1 & 2, client advised to choose best option…..4<br>Other (specify)______________________<br>_________________________________<br>______________________________….77	
3.8.6	In what circumstances would a woman be counseled to do exclusive breastfeeding? *[Circle all that apply}*	Infant formula not acceptable culturally…1<br>Stigma from formula use…2<br>Mother does not have time/skills/knowledge to use formula…3<br>Formula not affordable…4<br>No access to clean water…5<br>No regular access to formula…6<br>Mother unable to prepare fomula safely/hygienically…7<br>Nothing, it is covered in ANC/delivery care…..8<br>Other (specify)__________________...77	

3.8.7	If HIV positive women decide to breastfeed, when are they counseled to wean the infant?	At 6 months exactly…..1<br>Around about 6 months…..2<br>When the baby is ready…..3<br>Depends if infant is HIV positive…..4<br>Other time (specify):________________....77	
3.8.8	If ***infant*** is HIV positive, when are mothers counseled to stop breastfeeding?	At 6 months.….1<br>When the baby is ready.….2<br>After 1 year.….3<br>After 2 years.….4<br>Other time (specify):________________....77	
3.8.9	Does the baby get a physical exam at the post-partum visit?	Yes…..1<br>No….. 2<br>Don't know…..88	
3.8.10	Does the mother get a physical exam at the post-partum visit?	Yes…..1<br>No…..2	
3.8.11	Does the mother get offered an HIV test if she did not have one during pregnancy/delivery?	Yes…..1<br>No…..2	
3.8.12	Does the baby get an HIV test if the mother is positive?	Yes…..1<br>No….. 2	

3.8.13	Does the baby get an HIV test if mother's status is unknown?	Yes…..1<br>No…..2	
3.8.14	When is this infant first tested for HIV if mother is HIV positive or unknown status?	If the baby is symptomatic only…..1<br>_______weeks/months after birth	
3.8.15	What kind of HIV test is usually conducted on infants?	PCR test (viral load)…..1<br>HIV antibody (rapid/ELISA) test…..2<br>Don't know….88	

3.8.16	Is co-trimoxazole prophylaxis routinely given to infants at 6 weeks?	Yes…..1<br>No…..2	
3.8.17	Is a follow-up HIV test routinely conducted on infants, and if so when?	Test conducted at 1 year…..1<br>Test conducted at 18 months…..2<br>Other (specify)________________....77	
3.8.18	If the infant tests positive, what steps are taken?<br>*[Circle all that apply]*	CD4 count…..1<br>CD4 cell percent…..2<br>HAART initiated at clinic…..3<br>Infant referred for HAART (specify where_____________________)…..4<br>Infant followed up every month…..5<br>Other (specify)___________________...77	

3.8.19	Is there a system for tracking women in the PMTCT program if they do not come for post-partum follow-up?	Yes …..1<br>No….. .2	If no, go to 3.8.21
3.8.20	**If yes**, please describe:		
3.8.21	How often are HIV infected infants followed up?	Every month…..1<br>Every three months…..2<br>Other (specify)__________________....77	

3.8.22	What checks are routinely done for HIV infected infants?<br>*[Circle all that apply]*	Growth monitoring…..1<br>Check current illnesses…..2<br>Clinical examination…..3<br>Assess feeding difficulties…..4<br>Assess feeding patterns…..5<br>Nutritional support…..6<br>Free formula for those NOT breastfeeding…..7<br>Viral load testing…..8<br>Other (specify)________________________<br>________________________________....77	
3.8.23	Do you routinely ask about HIV status at well baby/immunization visits?	Yes…1<br>No…2	

3.8.24	What do you do if a woman's HIV status is unknown at well baby/immunization visit?	Test woman for HIV during visit…..1<br>Test baby for HIV during visit…..2<br>Test woman and baby for HIV during visit…..3<br>Refer woman and baby for HIV testing in this facility…..4<br>Refer woman and baby for HIV testing elsewhere…..5<br>Other (specify)________________________<br>________________________________.....77	

4. INTEGRATION OF HIV/SRH

All providers to respond.

	Question	Response
4.1	What comes to your mind when I say the phrase, "integrating HIV and SRH services"?<br>*[Circle all mentioned]*	One stop facility…..1<br>One stop provider…..2<br>Different services on different days within the same facility…..3<br>Comprehensive counseling.....4<br>Fast-tracking certain services…..5<br>Referral within facility…..6<br>Referral within the health system…..7<br>Referral between public and private health sectors…..8<br>Other (specify)__________________<br>______________________________<br>______________________________<br>__________________________….77
4.2	In your facility, what SRH Services are integrated so that a client can potentially be seen by the same Registered Nurse provider on the same day?<br>*[Read out and circle all that apply]*	FP…..1<br>Pap smear….. 2<br>STIs….. 3<br>VCT….. 4<br>PEP….. 5<br>Pregnancy testing….. 6<br>Sexual health counseling….. 7<br>Condom demonstration….. 8<br>ARV treatment….. 9<br>TB testing….10

If any services are not checked in the question above, list them under service column below, and ask the reason that they cannot be done by the same registered nurse.

4.3	Service	Reason for service not being done by same registered nurse

4.4	What do you see as some of the benefits, if any, of integrating sexual and reproductive health care into HIV care?	Improve quality of care…..1<br>Improve health of HIV patients…..2<br>HIV patients can get all services together…..3<br>Other (specify)__________________<br>______________________________<br>__________________________….77
4.5	What do you see as some of the challenges/barriers to integrating sexual and reproductive health care into HIV care?<br>*[Circle all mentioned]*	There is too much work to do for providers…..1<br>Staff do not have the training…..2<br>Staff find it uncomfortable to deal with HIV+ clients…..3<br>Other (specify) ___________….77
4.6	Do you think your facility is prepared to integrate sexual and reproductive health and HIV care in terms of record-keeping?	Yes….1<br>No…..2
4.7	Often services are very busy in the mornings and then few clients are seen in the afternoon. If logistics were addressed do you think health care staff would be prepared to see clients in the afternoon?	Yes …..1<br>No…..2<br>Not sure…..3
4.8	Do you think that a "one-stop shop" model of health care delivery would increase or decrease the time you spend on paperwork?	Increase paperwork…..1<br>Stay the same…..2<br>Decrease paperwork…..3
4.9	In your opinion, do you think your facility would be able to see all the patients that come each day to the facility if it became a one stop shop?	Yes…..1<br>No…..2<br>Already is a one-stop shop…..3<br>Not sure…..4
4.10	Do you think it would be a good idea for each client to see the same provider every time he or she came to the health care facility?	Yes….1<br>No…..2
4.11	What would have to change in the health care facility in order for this to be the case? Answer if yes or no above?	
4.12	Do you think that management here would be supportive of integrating HIV care and sexual and reproductive health?	Yes…..1<br>No…..2<br>We are already integrated…..3

5. ATTITUDE TOWARDS ARV AND FP

All providers to respond.

	**Read the given statement and circle the number on the scale that you feel best describe how you feel**	**1= strongly agree<br>2= somewhat agree<br>3= not sure<br>4= somewhat disagree<br>5= strongly disagree**				
5.1	Pregnancy and family planning are women's issues	1	2	3	4	5
5.2	Teenagers should abstain from sexual intercourse	1	2	3	4	5
5.3	HIV services will always be separate from MOPD because of the need for confidentiality	1	2	3	4	5
5.4	Woman on ARVs should not fall pregnant	1	2	3	4	5
5.5	Injectable contraceptives are safe for women taking ARVs	1	2	3	4	5
5.6	Married women with HIV will never use condoms	1	2	3	4	5
5.7	There is no point to use condoms together with another family planning method	1	2	3	4	5
5.8	Women who try and use condoms with their partner will be beaten	1	2	3	4	5
5.9	There is no point asking women to bring their partners to this facility because they will not come	1	2	3	4	5
5.10	All HIV positive women who fall pregnant should get a TOP	1	2	3	4	5
5.11	Providers who offer integrated care offer lower quality care than those who are specialist in one area	1	2	3	4	5
5.12	Infants who are HIV positive are not a priority for us	1	2	3	4	5

This is the end of our interview. Thank you so much for sharing your ideas with me. Do you have any questions, or is there anything that you would like to add before we end? If you have further thoughts about any of the issues we discussed today, please call Dr. Jenni Smit, the South African Principal Investigator of the study, her contact details are on the information sheet/consent form that you were given.
