# Supplementary material for: Fertility desires of people living with HIV: does the implementation of a sexual and reproductive health and HIV integration model change healthcare providers’ attitudes and clients’ desires?
Source: BMC Health Serv Res. 2021 May 26;21:509. doi: 10.1186/s12913-021-06487-0 (PMC8157636; doi:10.1186/s12913-021-06487-0)
Supplement: Supplementary file 2 — Additional file 2: Figure 1. Model diagram. [file 12913_2021_6487_MOESM2_ESM.docx]

Figure 1. Conceptual representation of SRH services integration model (37)

**Long term impact: Improved SRH & HIV outcomes**

**2. Health systems strengthening**

**3. Service level interventions**

Strengthen client & commodity monitoring

Strengthen referral & linkage systems

Strengthen existing SRH services

Support integration of FP and HIV services

**1. Capacity building: Training & mentorship**

**Outcomes**

Improved **practice**

(SRH & HIV service provision)

Change in **policy** on integrated services

4. Community input and involvement

4. Community input and involvement
